# *Pseudomonas aeruginosa* transcriptome analysis of metal restriction in *ex vivo* cystic fibrosis sputum

**DOI:** 10.1128/spectrum.03157-23

**Published:** 2024-02-22

**Authors:** Samuel L. Neff, Georgia Doing, Taylor Reiter, Thomas H. Hampton, Casey S. Greene, Deborah A. Hogan

**Affiliations:** 1 Department of Microbiology and Immunology, Geisel School of Medicine at Dartmouth, Hanover, New Hampshire, USA; 2 Department of Biomedical Informatics, University of Colorado School of Medicine, Aurora, Colorado, USA; University of Maryland School of Pharmacy, Baltimore, Maryland, USA

**Keywords:** cystic fibrosis, sputum, *ex vivo*

## Abstract

Chronic *Pseudomonas aeruginosa* lung infections are a feature of cystic fibrosis (CF) that many patients experience even with the advent of highly effective modulator therapies. Identifying factors that impact *P. aeruginosa* in the CF lung could yield novel strategies to eradicate infection or otherwise improve outcomes. To complement published *P. aeruginosa* studies using laboratory models or RNA isolated from sputum, we analyzed transcripts of strain PAO1 after incubation in sputum from different CF donors prior to RNA extraction. We compared PAO1 gene expression in this “spike-in” sputum model to that for *P. aeruginosa* grown in synthetic cystic fibrosis sputum medium to determine key genes, which are among the most differentially expressed or most highly expressed. Using the key genes, gene sets with correlated expression were determined using the gene expression analysis tool eADAGE. Gene sets were used to analyze the activity of specific pathways in *P. aeruginosa* grown in sputum from different individuals. Gene sets that we found to be more active in sputum showed similar activation in published data that included *P. aeruginosa* RNA isolated from sputum relative to corresponding *in vitro* reference cultures. In the *ex vivo* samples, *P. aeruginosa* had increased levels of genes related to zinc and iron acquisition which were suppressed by metal amendment of sputum. We also found a significant correlation between expression of the H1-type VI secretion system and CFTR corrector use by the sputum donor. An *ex vivo* sputum model or synthetic sputum medium formulation that imposes metal restriction may enhance future CF-related studies.

**IMPORTANCE:**

Identifying the gene expression programs used by *Pseudomonas aeruginosa* to colonize the lungs of people with cystic fibrosis (CF) will illuminate new therapeutic strategies. To capture these transcriptional programs, we cultured the common *P. aeruginosa* laboratory strain PAO1 in expectorated sputum from CF patient donors. Through bioinformatic analysis, we defined sets of genes that are more transcriptionally active in real CF sputum compared to a synthetic cystic fibrosis sputum medium. Many of the most differentially active gene sets contained genes related to metal acquisition, suggesting that these gene sets play an active role in scavenging for metals in the CF lung environment which may be inadequately represented in some models. Future studies of *P. aeruginosa* transcript abundance in CF may benefit from the use of an expectorated sputum model or media supplemented with factors that induce metal restriction.

## INTRODUCTION

*Pseudomonas aeruginosa* is a common cause of acute, hospital-acquired infections (1). Outside the hospital setting, chronic *P. aeruginosa* infections can occur in individuals with decreased immune-protective mechanisms including people with the genetic disease cystic fibrosis (CF) (2–5). In people with CF (pwCF), dysfunction of the anion transporter protein CFTR leads to the buildup of thick, sticky mucus in the lungs and other organs (6–8). Most pwCF experience defective mucociliary clearance and impaired defense against opportunistic pathogens (9, 10). Patients are susceptible to colonization by a broad range of bacterial and fungal pathogens (11–16). *P. aeruginosa* infection specifically tends to be more prevalent in older pwCF, where it is associated with reduced lung function, higher rates of hospitalization, and increased mortality (4, 5). Furthermore, antibiotic resistance is common in *P. aeruginosa* clinical isolates (17). Though recent advances in the treatment of CF (namely highly effective modulator therapies) have dramatically improved the expected life span, these treatments do not appear to eradicate established *P. aeruginosa* infections in most cases though they may reduce bacterial burden and virulence (18–21).

To understand how *P. aeruginosa* can persist in the CF lungs, it is important to acquire a more complete understanding of the transcriptional programs that govern the biological behavior of the bacterium. The biological behavior of any given *P. aeruginosa* isolate is determined by multiple factors. Genetic elements—intrinsic to the strain or acquired through horizontal gene transfer—can confer virulence traits or resistance to antibiotics (22–24). Additionally, *P. aeruginosa* virulence can be driven by aspects of the surrounding environment. Factors such as the composition of mucus or the community of other microbes in the lungs can have a major impact on *P. aeruginosa* phenotype, including virulence traits (25–28). These differences in phenotype can be associated with molecular profiles using high-throughput -omics techniques. For example, *P. aeruginosa* can modify its metabolic profile during the course of chronic lung infection, adopting specific “metabotypes” that are associated with increased virulence and antibiotic resistance (29). Likewise, researchers aim to draw associations between other -omics modalities (*P. aeruginosa* gene expression, protein expression, etc.) and virulence traits.

The metal acquisition activity of *P. aeruginosa* in the CF lungs has been a recent area of focus. Despite relatively high levels of metals such as zinc and iron in CF sputum compared to healthy individuals (30), several studies have shown that *P. aeruginosa* exhibits a zinc and iron restriction response due to the conditions of the CF lung (31–34). This counterintuitive phenomenon appears to be driven, at least in part, by the activity in CF sputum of human calprotectin, a metal-binding protein produced by neutrophils (31). Other metal sequestering molecules produced by the host or other microbial species in the CF lung play a role as well—for example, the *S. aureus* metallophore staphylopine, which competes for zinc and other metals (35, 36). Understanding the subtleties of the *P. aeruginosa* metal response—what genes are involved and what biological factors in the CF lung are driving their expression—is clinically important. Both zinc and iron intake have been associated with virulence traits like swarming motility and biofilm formation, as well as interaction with other CF pathogens (32, 37, 38). In addition, the concentrations of iron and zinc in sputum correlate inversely with clinical outcomes (30, 39). Further defining the transcriptomic signatures associated with this metal restriction response—as well as other transcriptomic signatures that define *P. aeruginosa* growth in CF sputum—could help predict patient clinical outcomes and illuminate new therapeutic strategies.

In this study, we sought to compare *P. aeruginosa* transcriptional profiles after growth in either an artificial sputum medium (40, 41) or in donated expectorated CF sputum. We used an experimental model in which the commonly used strain PAO1 was “spiked-in” to expectorated sputum and incubated prior to RNA extraction, sequencing, and RNA-seq analysis (31). The spike-in model has three intrinsic advantages over sequencing *P. aeruginosa* clinical isolates directly in CF sputum (i.e., gathering sputum from CF donors and sequencing the bacterial isolates that are present in the sputum directly). First, in the sputum of CF patients, the fraction of total extracted RNA from *P. aeruginosa* can be low and highly variable. This may impair the ability to detect and analyze lowly expressed *P. aeruginosa* genes. Second, the use of a single strain from a common inoculum allows for analysis of environmental conditions across sputa without the complication of strain-to-strain differences. Lastly, using the experimental model, we were able to directly manipulate the metal restriction response by comparing *P. aeruginosa* transcript abundance in sputum with and without the addition of iron, zinc, and manganese.

After setting up the experimental model and performing RNA sequencing, we analyzed differential gene expression and pathway activation to compare profiles of *P. aeruginosa* grown in CF sputum and synthetic cystic fibrosis sputum medium (SCFM2) (42) and further assessed how gene expression was affected by added metals. Notably, we found that genes related to zinc and iron acquisition have significantly higher transcript abundance in CF sputum than SCFM2 and that correlated gene sets containing these metal acquisition genes were repressed in a coordinated manner when the spike-in samples were treated with a mixture of metals. We further examined correlations between the average expression of all identified gene sets and donor characteristics like lung function (FEV1) and the use of different drugs. We report significant negative correlations between CFTR potentiator usage and the activity of specific gene sets, including one associated with the type VI secretion system, a well-known *P. aeruginosa* mechanism for microbe-microbe interactions. Relative to laboratory grown controls, *P. aeruginosa* transcriptomes from the *ex vivo* model compared favorably to profiles from *P. aeruginosa* RNA isolated from sputum published by Lewin et al. (43) with some of the differences likely being driven by differences in strain.

## RESULTS

### *Ex vivo* spike-in model for the analysis of *P. aeruginosa* transcriptomes in sputum

To determine if there are *P. aeruginosa* gene expression signals associated with exposure to CF sputum that are not adequately captured by laboratory media, we developed an *ex vivo* spike-in sputum model. In this model, *P. aeruginosa* strain PAO1 cultures were grown to mid-exponential phase (~0.5 OD_600_ nm) in M63 medium containing 0.2% glucose with high aeration and added zinc, iron, and manganese as described in the methods. The metal addition was sufficient to suppress the expression of metal acquisition genes in pilot studies. The *P. aeruginosa* cells were washed and concentrated to an OD_600nm_ of 10 prior to inoculation into sputum or SCFM2 medium (42).

This study includes the analysis of *P. aeruginosa* grown in sputum collected from 17 CF donors. The donors are distinguished by a variety of characteristics including whether they are on inhaled, oral, or IV antibiotics (and what kind they are on), whether they had been receiving CFTR potentiator therapy or not, which CF pathogens they had cultured at the time of the sample collection and which pathogens were repeatedly detected during the preceding 2 years (Table 1). For each sputum aliquot (>200 µL), the sample was homogenized and then divided into two 100 µL aliquots. One aliquot was amended with three metals (iron, zinc, and copper) at final concentrations of 300 µM ammonium ferrous sulfate, 150 µM zinc sulfate, and 10 µM manganese chloride. Other sputum samples received only water. Sputum aliquots and SCFM2 (also at a volume of 100 µL) were placed into 1.5-mL Eppendorf tubes, inoculated with OD 1 equivalent of *P. aeruginosa* in 10 µL, and incubated with gentle agitation and open caps in a humidified chamber for 3 h (see Materials and Methods). Total RNA was extracted and processed via Salmon as described previously (44, 45).

**TABLE 1:** Clinical characteristics of CF donors^*a*^

Subj. ID	FEV1	Antibiotic usage	Potentiator therapy	Sputum microbiology details	Reported chronic infections (2 years)
105	88	No inhaled abx; No oral abx; IV abx (ceftazidime, tobramycin)	No	*Pa* (mucoid and nonmucoid), *Candida albicans*	*Pa* (mucoid and nonmucoid), *Candida albicans*
106	35	Inhaled abx (cayston); oral abx unknown; IV abx unknown	N/A	N/A	*Burkholderia* sp.
108	38	Inhaled abx (colistin); oral abx (azithromycin, other); IV abx (meropenem)	No	*Burkholderia* sp., *C. albicans*	*Burkholderia* sp.
120	22	Inhaled abx (cayston); oral abx (azithromycin); IV abx (ceftazidime)	Yes	*Pa* (mucoid and non-mucoid) *Burkholderia* sp.	*Pa* (mucoid and non-mucoid) *Burkholderia* sp.
122	86	No inhaled abx; oral abx (other); IV abx (ceftazidime)	No	MSSA, *C. albicans*, *Aspergillus* sp.	MSSA, *C. albicans*, *Aspergillus* sp.
124	91	Inhaled abx (cayston, other); oral abx (doxycycline, other); no IV abx	Yes	*Pa* (mucoid and nonmucoid), MSSA, *C. albicans*	*Pa* (mucoid and nonmucoid), MSSA, *C. albicans*; *Aspergillus* sp.
125	38	No inhaled abx; oral abx (azithromycin); IV abx (meropenem, tobramycin)	No	*Pa* (mucoid), *C. albicans*	*Pa* (mucoid), *C. albicans*
133	68	No inhaled abx; oral abx (other); IV abx (other)	Yes	*Candida* sp.	*Candida* sp.
138	45	Inhaled abx (cayston, other); no oral abx; no IV abx	Yes	*Pa* (mucoid and nonmucoid), *C. albicans*	*Pa* (mucoid and nonmucoid), *C. albicans*
145	34	Inhaled abx (tobramycin); no oral abx; no IV abx	Yes	*Pa* (mucoid), MRSA	*Pa* (mucoid), MRSA; *Aspergillus* sp.
153	86	No inhaled abx; no oral abx; IV abx (ceftazidime, vancomycin, tobramycin)	Yes	MRSA, *Candida* sp.	MRSA, *Candida* sp.
201	56	No inhaled abx; oral abx (ciprofloxacin); IV abx (other)	No	MRSA	MRSA
203	64	Inhaled abx (tobramycin); oral abx (azithromycin); IV abx (ceftazidime)	No	*Pa* (mucoid)	*Pa* (mucoid); *C. albicans*; *Candida* sp.
204	48	No inhaled abx; no oral abx; IV abx (ceftazidime, tobramycin)	Yes	*Pa* (mucoid), *C. albicans*	*Pa* (mucoid), *C. albicans*
220	69	No inhaled abx; oral abx (azithromycin); No IV abx	Yes	*Pa* (nonmucoid), MSSA	*Pa* (nonmucoid), MSSA
233	22	Inhaled abx (tobramycin, cayston); oral abx (azithromycin, ciprofloxacin, other); IV abx (meropenem, vancomycin)	No	*Pa* (mucoid and nonmucoid), MRSA, *Bulkholderia* sp.	*Pa* (mucoid and nonmucoid), MRSA, *Bulkholderia* sp.
239	53	Inhaled abx (cayston); oral abx (azithromycin); IV abx (ceftazidime, tobramycin)	Yes	*Pa* (mucoid), *Aspergillus* sp.	*Pa* (mucoid), *Aspergillus* sp.
243	45	Inhaled abx (other); oral abx (other); IV abx (ceftaroline)	Yes	MRSA, *C. albicans*	MRSA, *C. albicans*

^*a*^ FEV1, percent predicted forced expiratory volume in one second; Abx, antibiotics; IV, intravenous; *Pa, P. aeruginosa*; MSSA, methicillin-sensitive *S. aureus*; MRSA, methicillin-resistant *S. aureus*; N/A, not available; reported chronic infections (2 years), microbes that were repeatedly detected over 2 years. Donor 102 provided gene expression data and is identified in subsequent figures but does not have associated metadata and, therefore, is not represented in this table.

### PAO1 transcript abundance in the spike-in sputum model systematically differs from artificial sputum medium

**Fig 1:**
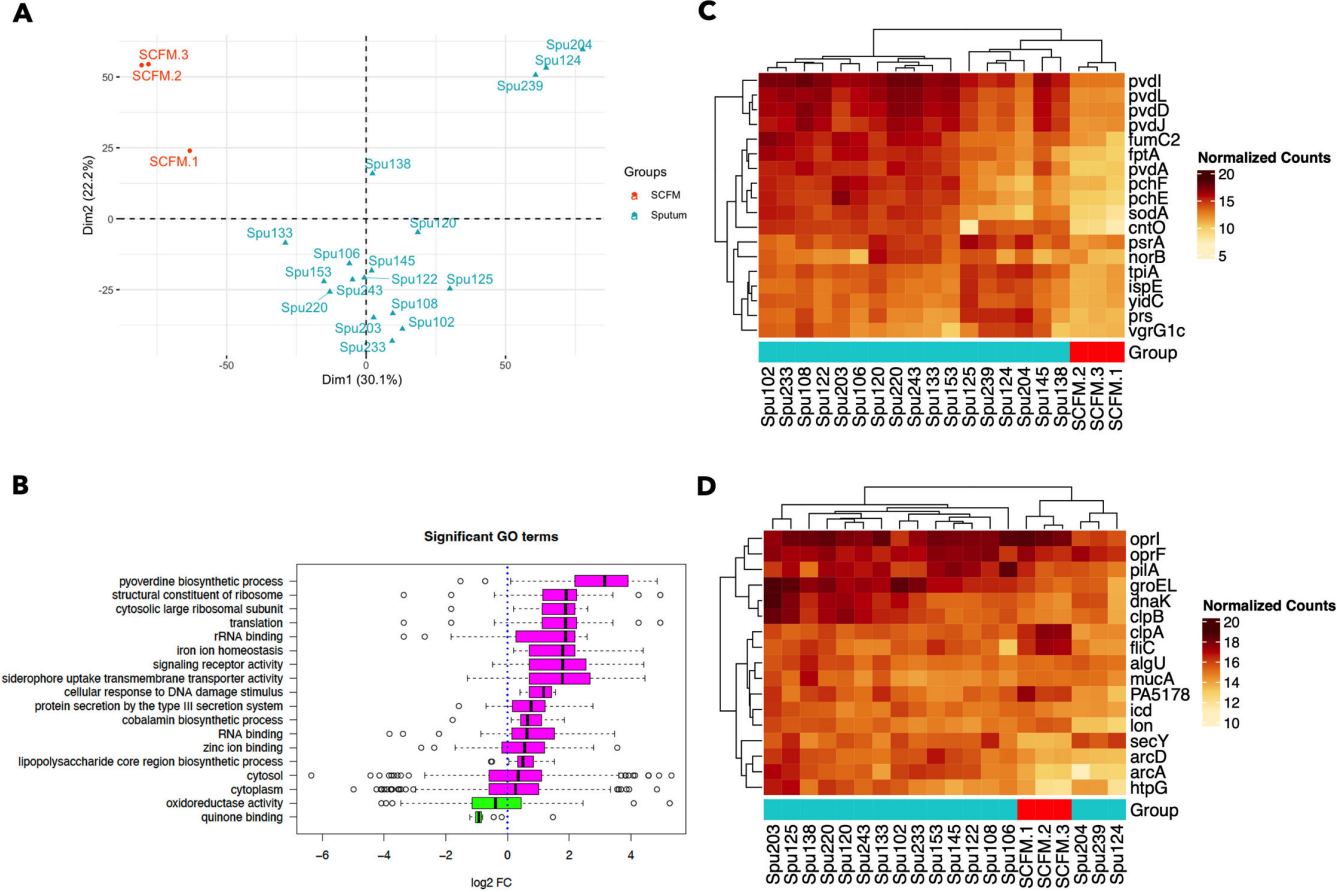
Transcriptional profiles of *P. aeruginosa* strain PAO1 after incubation in CF sputum or SCFM2. (**A**) PCA, considering all genes detected by RNA sequencing, clusters the CF sputum and SCFM2 samples separately, with most CF sputum samples clustering closely together. (**B**) The pathway activation analysis identified 18 significantly activated or repressed GO terms in expectorated CF sputum compared to SCFM2. Pink pathways are activated in CF sputum compared to SCFM2 and green pathways are repressed (**C**) All key genes listed in Table 2 that were identified as highly differentially expressed between the CF sputum and SCFM2 samples were elevated in their expression across most of the CF sputum samples compared to SCFM2. (**D**) The key genes that were identified for their high average expression across *P. aeruginosa* grown in different CF sputum samples are also shown, demonstrating further differences between the three outlier sputum samples (Spu124, Spu204, Spu239) and the rest of the spike-in sputum samples, as well as the SCFM2 samples. Heat map panels C and D present log_2_-transformed and normalized count levels, as indicated in the legends.

First, we performed differential gene expression analysis to understand how the transcriptomes of *P. aeruginosa* strain PAO1 in CF sputum compared to those for cells grown in SCFM2. Analysis of the PAO1 transcriptome in CF sputum compared to SCFM2 found 2,364 genes that were significantly differentially expressed (FDR corrected *P* value < 0.05) in CF sputum compared to SCFM2 (Table S1) (46). Furthermore, principal component analysis (PCA) clearly distinguished the SCFM2 and spike-in CF sputum samples (Fig. 1A). Most of the spike-in sputum samples clustered relatively closely, as did the SCFM2 samples, though three sputum samples (from donors 124, 204, and 239) were quite distinct from both the other spike-in samples and the SCFM2 samples (Fig. 1A).

Pathway activation analysis was performed with the edgeR differential expression data as input using the web application ESKAPE Act Plus. This form of pathway analysis is based on the binomial test. Assuming that positive and negative fold changes in gene expression are equally likely, the statistical significance of pathway activation/repression is based solely on the proportion of genes with positive or negative fold changes, not the proportion of genes with a FDR-corrected *P* value less than 0.05. Thus, a significantly “activated” pathway will have a higher proportion of genes with a positive fold change than expected by chance, while a significantly “repressed” pathway will have a higher proportion of genes with a negative fold change than expected by chance (47). This approach contrasts with the “gene set enrichment analysis” (GSEA) approach where genes are ranked by their fold change and significiance of differential expression, and pathways are deemed “enriched” when a high proportion of genes (compared to the proportion expected by chance) are found at either the top or the bottom of this ranked list. Given the relatively large total number of significantly differentially expressed genes in this study (see discussion of limitations below), we opted for a pathway activation analysis approach.

Pathway activation analysis identified 18 functional pathways (GO terms) that were significantly activated or repressed between the SCFM2 and spike-in sputum samples with an FDR-corrected *P* value < 0.05. Compared to culture in SCFM2, PAO1 grown in CF sputum exhibited elevated expression of genes related to metal acquisition (GO terms: pyoverdine biosynthesis, iron ion homeostasis, siderophore uptake transmembrane transporter activity, zinc ion binding). In addition to metal acquisition signatures, PAO1 growth in CF sputum is also distinguished from SCFM2 by the activation of GO terms related to LPS biosynthesis, vitamin B biosynthesis, type III secretion, and the DNA damage response (Fig. 1B).

Beyond characterizing the broad differences between sample groups, we narrowed a much shorter list of “key genes” that characterize PAO1 transcript abundance in CF sputum on the basis of differential expression, abundance, and involvement in processes other than translation/protein synthesis, cell division, or ATP synthesis (Table 2). For the prioritization of genes by differential expression, we first identified the genes that were most highly differentially expressed in the spike-in sputum samples compared to SCFM2. This involved first taking the subset of genes with a positive fold difference and then identifying genes in this subset with a fold change value in the top 10%, a logCPM value in the top 10%, and an uncorrected *P* value < 0.05 (all three conditions had to be met at once for a gene to be considered a “key gene”). To identify key genes by high expression in CF sputum, even if they are also highly expressed in SCFM2, the 50 genes with the highest average count values across just the spike-in CF sputum samples were included. Lastly, genes involved in translation, cell division, or ATP production (60 genes in total) were removed from the list of genes for this analysis to focus on genes that are involved in functions beyond cellular maintenance, but it is acknowledged that the analysis of expression of these genes may yield important information. These excluded genes are described further in the methods and included in Table S2 and are available for future analyses. The above method for gene prioritization yielded 35 key genes (Table 2).

**TABLE 2:** Key genes that describe PAO1 transcripts most strongly differentially expressed in the spike-in sputum model compared to when grown in SCFM2 and/or genes most highly expressed in *P. aeruginosa* grown in *ex vivo* sputum

Locus ID	Gene name	Protein name	High DE (sputum vs SCFM2)	High ave expression in sputum
PA4669	*ispE*	4-Diphosphocytidyl-2-C-methyl-D-erythritol kinase	Y	N
PA5171	*arcA*	Arginine deiminase	N	Y
PA5170	*arcD*	Arginine/ornithine antiporter	N	Y
PA2620	*clpA*	ATP-binding protease component	N	Y
PA1092	*fliC*	B-type flagellin	N	Y
PA4542	*clpB*	Chaperone protein	N	Y
PA4761	*dnaK*	Chaperone protein DnaK (HSP70)	N	Y
PA4385	*groEL*	Chaperonin	N	Y
PA4221	*fptA*	Fe(3+)-pyochelin receptor	Y	N
PA4470	*fumC2*	Fumarate hydratase	Y	Y
PA1596	*htpG*	Heat shock protein	N	Y
PA2623	*icd*	Isocitrate dehydrogenase	N	Y
PA1803	*lon*	Lon protease	N	Y
PA2853	*oprI*	Major outer membrane lipoprotein	N	Y
PA5568	*yidC*	Membrane protein insertase	Y	N
PA0524	*norB*	Nitric oxide reductase	Y	N
PA1777	*oprF*	Outer membrane porin	N	Y
PA5178	*PA5178*	Peptidoglycan-binding protein LysM	N	Y
PA4243	*secY*	Protein translocase	N	Y
PA4837	*cntO*	Pseudopaline receptor (Zn uptake)	Y	N
PA4226	*pchE*	Pyochelin synthase	Y	N
PA4225	*pchF*	Pyochelin synthase	Y	N
PA2424	*pvdL*	Pyoverdine biosynthesis	Y	Y
PA2386	*pvdA*	Pyoverdine biosynthesis	Y	N
PA2402	*pvdI*	Pyoverdine biosynthesis	Y	Y
PA2400	*pvdJ*	Pyoverdine biosynthesis	Y	Y
PA2399	*pvdD*	Pyoverdine synthetase	Y	Y
PA4670	*prs*	Ribose-phosphate pyrophosphokinase	Y	N
PA0762	*algU*	RNA polymerase sigma factor	N	Y
PA0763	*mucA*	Sigma factor AlgU negative regulatory protein	N	Y
PA4468	*sodA*	Superoxide dismutase	Y	N
PA3006	*psrA*	Transcriptional regulator	Y	N
PA4748	*tpiA*	Triosephosphate isomerase	Y	N
PA4525	*pilA*	Type IV major pilin protein PilA	N	Y
PA2685	*vgrG1c*	Type VI secretion system spike protein	Y	N
				

Of 18 key genes that were identified based on increased expression in the donated sputum samples relative to expression in cells grown in SCFM2 (Fig. 1C), the most notable characteristic was an association with metal acquisition, including the gene encoding the zincophore pseudopaline receptor *cntO*, which is implicated in zinc uptake (48), and iron acquisition genes including the pyochelin receptor *fptA* and pyochelin and pyoverdine synthesis genes (*pchE*, *pchF*, *pvdA*, *pvdD*, *pvdJ*, *pvdL*) (49, 50). The expression of *sodM* and *fumC1* has also been shown to be regulated as part of a response to iron limitation particularly in cells with high AlgU activity (51). The elevated expression of these genes in donated CF sputum suggests that *P. aeruginosa* is experiencing conditions of metal restriction, as previous studies have suggested in studies with smaller sets of sputum samples and using different approaches (31–34). The additional implication of the results in this study is that this metal restriction phenotype is not displayed, or at least not displayed as acutely, in SCFM2. Other key genes that are more highly expressed in CF sputum are discussed in more detail below.

Seventeen of the 35 key genes identified were selected by high abundance in sputum (Table 2; Fig. 1D). Of these, all but four were also among the highest average expression (in the top 10% of genes by average expression) in a separate normalized compendium of 890 RNA-seq samples for *P. aeruginosa* strain PAO1 grown in diverse conditions in different labs and with different engineered mutations that we published previously, suggesting that they were generally highly expressed (44). The four genes that had high expression in sputum that were not among the top 10% of genes by average expression in the PAO1 RNA-seq compendium were *algU*, *mucA*, *htpG* (heat shock response protein), and *PA5178* (peptidoglycan-binding protein). The high expression of the sigma factor-encoding gene *algU*/*algT* and *mucA*, which encodes an anti-sigma factor that regulates AlgU, is interesting in light of the frequent *mucA* mutations observed in *P. aeruginosa* isolates from CF-related lung infections (52, 53). AlgU has also been implicated in the response to oxidative stress and a variety of other stressors. Indeed, prior studies have noted that the hyperinflammatory state of the CF lungs generates conditions of oxidative stress, which has been associated with the prevalence of hypermutable, antibiotic-resistant *P. aeruginosa* isolates (54, 55).

There was some degree of variation in PAO1 key gene expression across the CF sputum samples (Fig. 1C and D). Heat map analysis of the normalized expression of key genes (as counts per million, CPM) that are more highly expressed in CF sputum relative to SCFM (Fig. 1C) found that samples clustered into several distinct groups. Most distinct are the three sputum samples (Spu124, Spu204, Spu239) that are also clustered distinctly in the PCA plot (Fig. 1A). These samples exhibited decreased expression of metal acquisition genes relative to the other spike-in samples, and increased expression of certain other key genes (*ispE*, *yidC*, *tpiA*, *prs*, and *vgrG4*). Several other sputum samples (Spu125, Spu138, Spu145) also stood out for their reduced expression of metal acquisition genes though the difference is less stark. Figure panel 2D, which includes genes characterized by high average expression across the CF sputum samples, further distinguished outlier samples Spu124, Spu204, and Spu239 from the other sputum samples in terms of their gene expression profile. There were no obvious differences in donor or sputum characteristics (Table 1) that could explain these differences.

### Identifying correlated gene sets that distinguish PAO1 gene expression in real CF sputum and SCFM

Analysis of the *P. aeruginosa* transcriptome in CF sputum from different donors can also reveal sets of genes with correlated expression. Correlated gene sets may be part of the same operon or regulon and/or simply responsive to the same external conditions. Like biological pathways (e.g., GO terms), these correlated gene sets may be more or less active as a unit between different groups of samples.

To identify correlated gene sets, we used the key genes that we identified as characteristic of *P. aeruginosa* strain PAO1 when grown in CF sputum (Table 2) to define gene sets containing other genes with highly correlated expression patterns. Specifically, we used the ADAGE web application to identify correlated gene sets (56, 57). The application is based on eADAGE, a neural network model trained on a compendium of over 1,000 *P*. *aeruginosa* microarray data sets generated by different labs for different purposes. The eADAGE model has been used previously to identify robust gene expression patterns (56, 57). Many gene sets identified by ADAGE contained genes within a common KEGG pathway and often genes within the same operon were part of the same gene set (Table 3).

**TABLE 3:** Core genes and associated gene ontology (GO) Terms for gene sets

Gene set	Core gene(s)	Associated GO terms
1	*cntO*	Zinc ion transport [GO:0006829]
2	*sodA*	Removal of superoxide radicals [GO:0019430], siderophore transport [GO:0015891], heme oxidation [GO:0006788]
3	*pchE, fptA, pchF*	Pyochelin biosynthetic process [GO:0042864]
4a6	*pvdA, pvdL, pvdI, pvdD, pvdJ*	Pyoverdine biosynthetic process [GO:0002049]
5	*fumC2*	Tricarboxylic acid cycle [GO:0006099]
7	*prs, secY*	Ribonucleoside monophosphate biosynthetic process [GO:0009156]; protein transport by the Sec complex [GO:0043952]; chaperone-mediated protein folding [GO:0061077]
8	*tpiA*	Glycolytic process [GO:0006096]; gluconeogenesis [GO:0006094]; glycerol catabolic process [GO:0019563]; protein secretion [GO:0009306]
9	*vgrG1c*	Protein secretion by the type VI secretion system [GO:0033103]
10	*norB*	Denitrification pathway [GO:0019333]
11	*ispE*	Terpenoid biosynthetic process [GO:0016114]
12	*yidC*	Protein transport by the Sec complex [GO:0043952]; lipopolysaccharide transport [GO:0015920]
13	*psrA*	Fatty acid beta-oxidation [GO:0006635]; electron transport chain [GO:0022900]
14	*oprI*	Lipid A biosynthetic process [GO:0009245], DNA recombination [GO:0006310]; positive regulation of transcription [GO:0045893]; regulation of translation [GO:0006417]
15	*oprF*	Ribosome disassembly [GO:0032790], cell division [GO:0051301]; protein polymerization [GO:0051258]
16	*groEL mopA, dnaK, htpG, clpB*	Chaperone cofactor-dependent protein refolding [GO:0051085]; protein folding [GO:0006457]; protein quality control for misfolded or incompletely synthesized proteins [GO:0006515]
17	*pilA*	Type IV pilus-dependent motility [GO:0043107]; DNA restriction-modification system [GO:0009307]
18	*algU, mucA*	Cellular response to cell envelope stress [GO:0036460] or oxidative stress [GO:1902884]; alginic acid biosynthetic process [GO:0042121] bacterial-type flagellum-dependent swarming motility [GO:0071978]; type IV pilus-dependent motility [GO:0043107]
19	*clpA*	Protein unfolding [GO:0043335]; proteolysis [GO:0006508]
20	*arcD, arcA*	Arginine deiminase pathway [GO:0019546]; lipopolysaccharide core region biosynthetic process [GO:0009244]
21	*fliC*	Bacterial-type flagellum-dependent cell motility [GO:0071973]
22	*PA5178*	Regulation of gene expression [GO:0010468], response to potassium ion [GO:0035864],cellular response to antibiotic [GO:0071236]; regulation of nitrogen utilization [GO:0006808]
23	*icd*	FtsZ-dependent cytokinesis [GO:0043093]; tricarboxylic acid cycle [GO:0006099]; regulation of RNA stability [GO:0043487]
24	*lon*	Chaperone cofactor-dependent protein refolding [GO:0051085]; protein refolding [GO:0042026]; intracellular iron ion homeostasis [GO:0006879]

For each of the 35 key genes, ADAGE was used to determine the 10 genes (including the key gene) that are best correlated in gene expression. If ADAGE identified fewer than 10 correlated genes across its associated compendium, all correlated genes were included. If two gene sets had 5 or more correlated genes in common, they were consolidated into a single gene set—bringing the total number of gene sets down to 24 (Table S2). Gene sets 4 and 6, though not meeting our criteria for consolidation, contained genes that are part of the same *pvd* operon. Thus, they were combined into a single gene set. For all but one gene set (gene set 9), all genes that were identified as correlated in expression by ADAGE had a Pearson correlation coefficient relative to the key gene of 0.5 or greater, and they were also positively correlated in their expression across our spike-in sputum samples. Component genes within gene sets were part of common processes as shown by mapping to one or several related “GO biological process” terms using the Uniprot ID Mapping Tool. A small number of genes were present in multiple different gene sets as indicated (Table S2). After identifying and characterizing individual gene sets, we analyzed the activity of each gene set for each sample’s transcriptome using the average normalized gene expression for all the genes in each gene set. This yielded a matrix of average gene expression values for each gene set in each sample (Table S3). which could be compared across samples based on z-scores (Fig. 3A). The median value for each gene set for samples from SCFM2 and sputum was also determined (Table S4).

The *P. aeruginosa* gene sets that were differentially active in sputum compared to SCFM included those related to zinc and iron acquisition (1, 2, 3, and 4a6) and also includes gene sets 7, 8, 11, 12, and 13, related to nucleotide biosynthesis, sugar biosynthesis, terpenoid biosynthesis, protein transport, LPS transport, and fatty acid oxidation respectively (see Table S2 for genes within gene sets). In contrast, there were five gene sets where average expression is quite high in SCFM relative to CF sputum. This includes gene sets 5, 17, 19, 21, and 22, with functional associations including the TCA cycle, type IV pilus-dependent motility, proteolysis, flagellar motility, and the potassium response, respectively.

**Fig 2:**
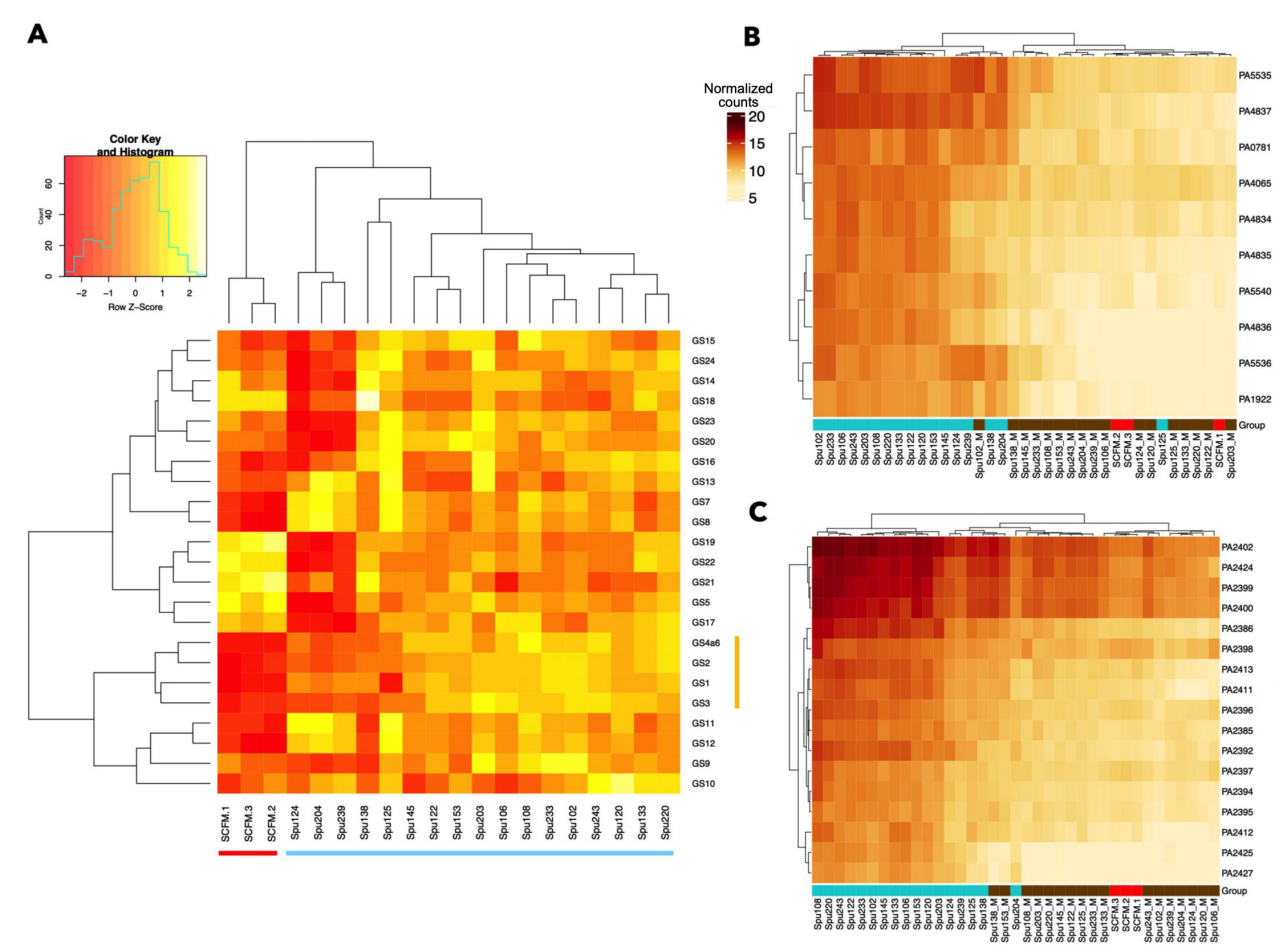
The average expression of each correlated gene set relating to metal acquisition across the spike-in sputum and SCFM2 samples. (**A**) Gene sets related to zinc and iron acquisition (1, 2, 3, 4, and 6), nucleotide (7), sugar (8), terpenoid biosynthesis (11), and fatty acid oxidation (13) were consistently elevated in the CF sputum samples compared to SCFM2. The SCFM2 samples (red) and sputum samples (light blue) are indicated along the bottom and metal acquisition-related gene sets (yellow) are indicated along the right side of the heat map in panel A. (**B, C**) Panels B and C demonstrate that for gene sets 1 (**B**) and 4a6 (**C**) the metal-treated and non-treated spike-in samples are clearly distinguished in terms of their gene expression at the individual gene level. In panels B and C, a darker red color indicates higher gene expression, while lighter orange/beige colors indicate reduced expression. Metal-treated samples are underlined brown. Versions of these heat maps for all 24 gene sets are included in Fig. S1.

We found that the 14 CF sputum transcriptomes that clustered together in the PCA plot (Fig. 1A) also clustered together by gene set activity (Fig. 2A). The three “outlier” sputum samples (Spu124, Spu204, Spu239) clustered distinctly in this heatmap as they did in Fig. 1A. They are distinguished from the other 14 sputum samples mainly by the heightened average expression of gene sets 7, 8, 11, 12, and 13 (functional associations including nucleotide biosynthesis, sugar biosynthesis, terpenoid biosynthesis, protein transport, LPS transport, and fatty acid oxidation), the diminished average expression of gene sets 5,17,19, 21, and 22 (functional associations including the TCA cycle, type IV pilus-dependent motility, proteolysis, flagellar motility, and the potassium response), as well as gene sets 14, 15, 16, 18, 20, 23, and 24 (functional associations including LPS biosynthesis, oxidative stress response, alginate biosynthesis, swarming motility, type IV pilus-dependent motility, and the TCA cycle) relative to the main cluster of CF sputum samples.

### Metal exposure drives the expression of gene sets linked to metal acquisition

The increased activity of gene sets related to metal acquisition in CF sputum relative to SCFM2 led us to hypothesize that the conditions of the CF lung deprive *P. aeruginosa* of metal and induce a metal restriction response that is not represented in the transcriptional profile of *P. aeruginosa* grown in SCFM2. In an earlier study, using a similar spike-in sputum model with PAO1, we had demonstrated that expression of genes related to zinc acquisition was significantly elevated in CF sputum compared to M63 minimal medium and that addition of metals to CF sputum suppressed the expression of these genes near the levels observed in the M63 medium-grown cells (31). Here, we performed a similar experiment to assess the effects on gene expression of supplementing sputum with a mixture of zinc, iron, and manganese (as described in the Materials and Methods) prior to *P. aeruginosa* strain PAO1 incubation.

**Fig 3:**
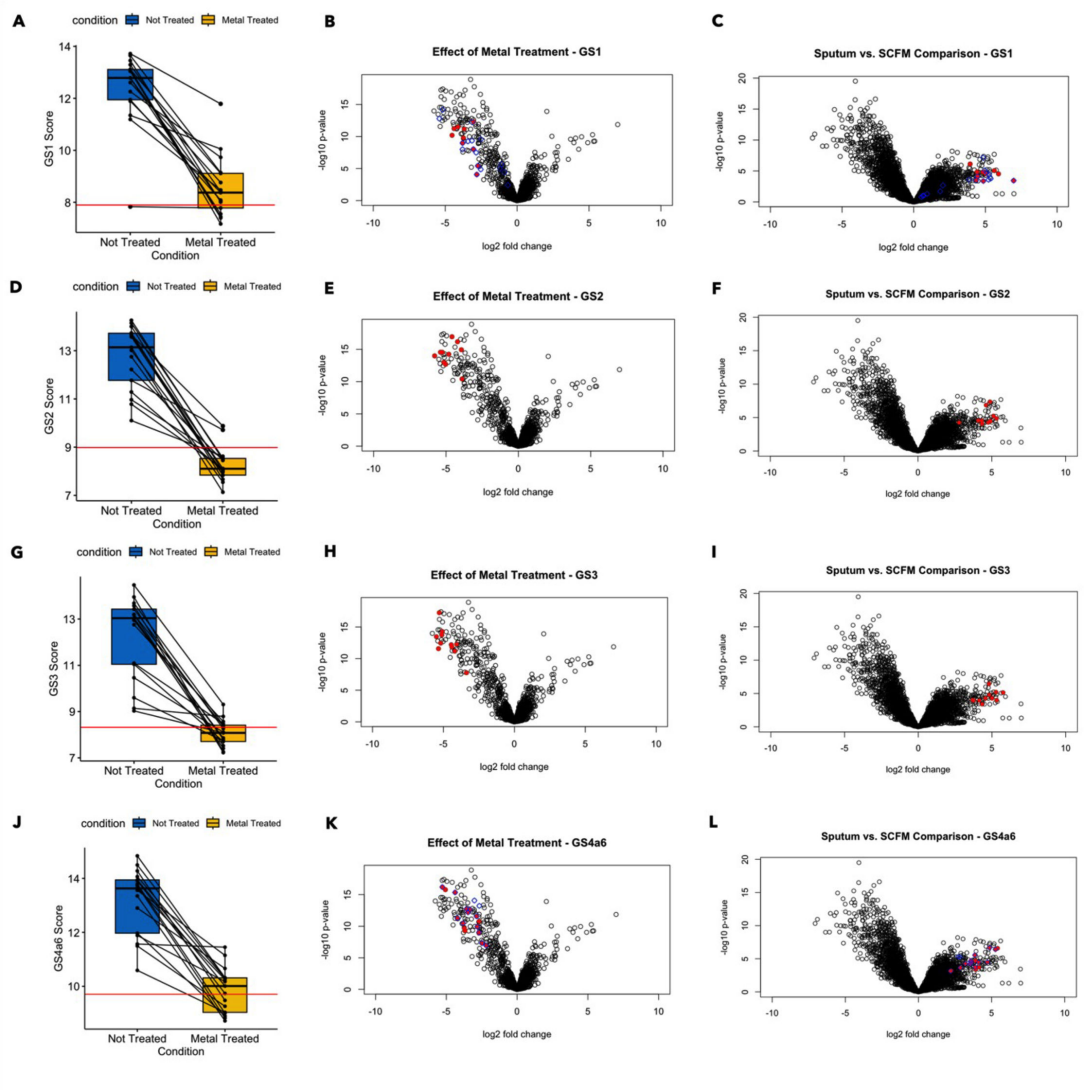
Effects of metals on *P. aeruginosa* transcript levels in *ex vivo* sputum. Treatment with a metal mixture containing zinc and iron repressed activation of metal acquisition gene sets in a consistent and coordinated manner. For the four gene sets depicted, the spike-in sputum samples saw a substantial reduction in overall gene set activation after metal treatment. In each case, the median value for average gene set expression across the spike-in samples was brought closer to the median value for the three SCFM2 samples after metal treatment (**A, D, G, J**). At the individual gene level, all genes incorporated in the four gene sets saw their expression significantly reduced by metal treatment (FDR < 0.05, |Fold Change| > 4) (**B, E, H, K**). All individual genes had initially been significantly more highly expressed in CF sputum vs SCFM2 (**C, F, I, L**). The blue circles overlaid onto panels B and C represent the Zur regulon, while the blue circles overlaid onto panels K and L represent the *pvd* regulon. We further analyzed associations between the average expression of the metal acquisition gene sets and donor clinical parameters. There were slight negative associations between average gene set expression and donor FEV1 for each of the metal acquisition gene sets though none of these associations were statistically significant (Fig. S4). There were also significant negative associations between IV tobramycin use and the average expression of gene sets 1, 2, 4, and 6. Though the association did not remain significant after FDR correction, it is possible that IV tobramycin treatment could increase metal availability to *P. aeruginosa* or suppress the activity of *P. aeruginosa* metal acquisition genes by some other mechanism (Fig. S5).

First, focusing on the expression levels of individual genes involved in zinc acquisition (gene set 1; Fig. 2B) and iron acquisition (gene set 4a6; Fig. 2C), we found that *P. aeruginosa* had lower expression of metal acquisition-associated genes in metal-treated samples than in untreated CF sputum, and the samples that received metal treatment clustered together. Analyzing the effects of added metals on gene set activity, we found that all of the gene sets related to metal acquisition were responsive to metal treatment, and again metal-treated and non-treated samples clustered distinctly in terms of the expression of their individual genes (Fig. S1). When *P. aeruginosa* expression in metal-treated and untreated samples from each donor was compared, all the individual genes in these gene sets were repressed to a statistically significant degree in the metal-treated samples. For the four gene sets involved in zinc acquisition (gene set 1) and iron acquisition (gene sets 2, 3, and 4a6), metal exposure reduced gene set activity. This effect was observed at the level of overall expression: the average expression of all four metal acquisition gene sets was elevated in non-treated samples compared to metal-treated samples (Fig. 3A, D, G, and J). The paired plots in Fig. 3-panels A, D, G, and J further indicate that for each individual donor, metal exposure had the effect of reducing average gene expression (though for some donors, this effect was more dramatic than others). The effects of metal exposure are also visible at the level of individual gene expression. We performed an additional differential gene expression analysis with edgeR, comparing the collection of CF spike-in sputum samples treated with metals to those not treated with metals. Every one of the constituent genes in gene sets 1–4a6 saw their expression reduced at least four-fold with an FDR-corrected *P* value < 0.05 (Fig. 3-panels B, E, H, K). None of the other gene sets exhibited a consistent, correlated response to metal treatment (Fig. S2A).

**Fig 4:**
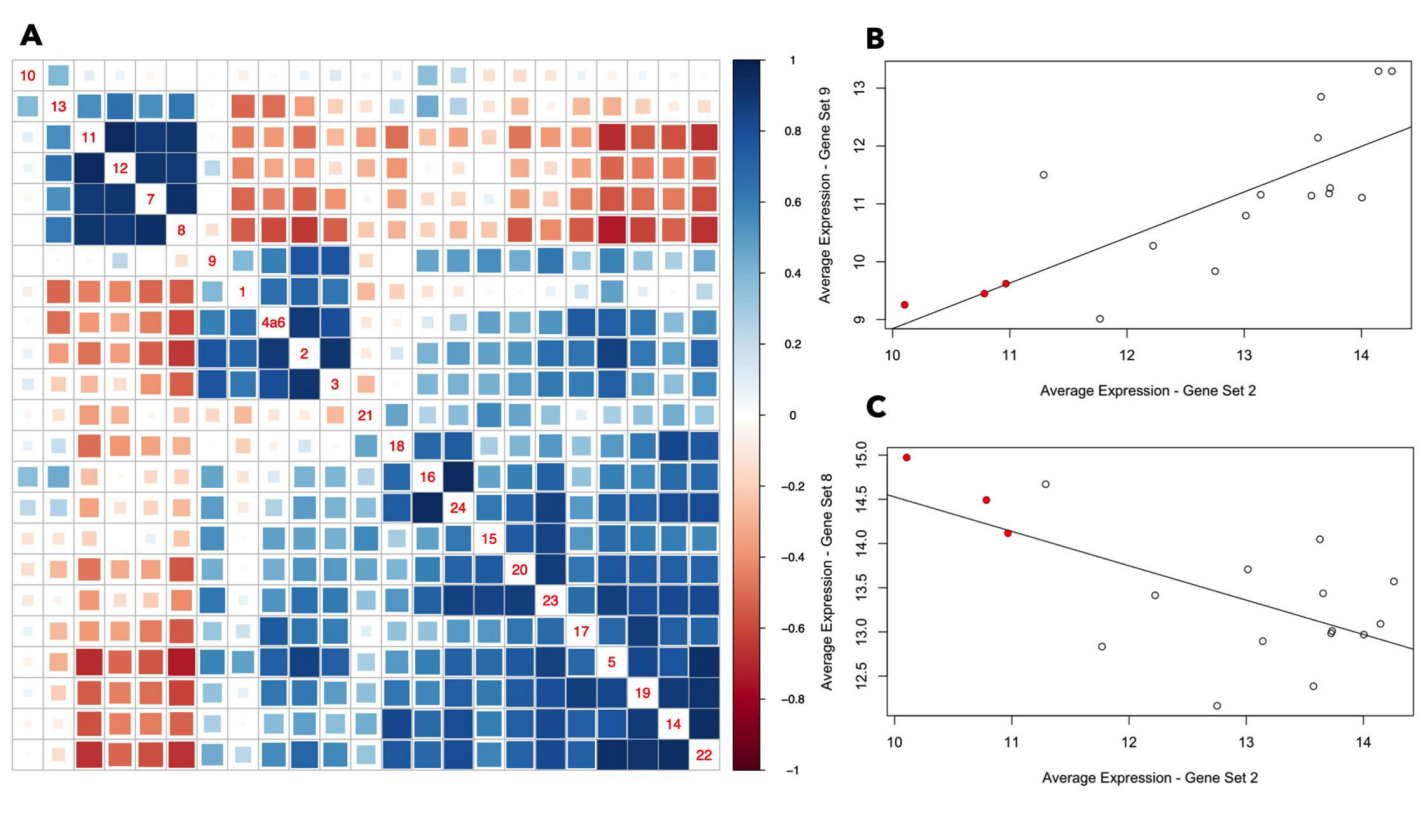
Correlation of average expression of gene sets across the spike-in CF sputum samples. (**A**) Gene sets 7, 8, 11, 12, and 13 are anti-correlated in their average expression across samples with most of the other gene sets (which are generally well correlated). Gene set 10 is an outlier in that it is not particularly well correlated with any of the other gene sets. Two gene sets with a Pearson correlation closer to +1 are represented by a darker blue box, while two gene sets with a Pearson correlation closer to −1 are represented by a darker red box. The gene set numbers are indicated along the diagonal, and each colored box is associated with two gene sets—for example, the box four up from the bottom in the right-most column indicates the correlation between gene sets 5 and 22. (**B, C**) Examples of gene set pairs that are (**B**) correlated and (**C**) anti-correlated. The red dots represent the three outlier samples (Spu124, Spu204, Spu239) referenced earlier in the manuscript.

For all four gene sets shown in Fig. 4, metal supplementation reduced gene set activity in CF sputum so that it was closer to the level of gene set activity seen in SCFM2. The red line in Fig. 3 panels A, D, G, and J represents the median value for the average gene set expression across the three SCFM2 samples. Figure 3 panels C, F, I, and L further show that all the individual genes in gene set 4 are significantly more active in CF sputum vs SCFM2.

Two additional observations reinforce the finding that the metal acquisition response is elevated in CF sputum compared to SCFM2. First, pathway activation analysis found that GO terms related to metal acquisition (“siderophore uptake transmembrane transporter activity,” “pyoverdine biosynthetic process,” “regulation of iron ion transport”) were significantly repressed when the spike-in sputum samples were exposed to the metal mixture (Fig. S3; Table S5). Second, genes from the established Zur regulon (blue circles overlaid onto Fig. 3 panels B and C) and PvdS regulons (blue circles overlaid onto panels 3K and 3L), which overlap with certain genes in gene sets 1 and 4, respectively, are also more active in CF sputum vs SCFM2 and repressed by metal exposure.

Finally, we examined the correlation between total sample metal concentrations in CF sputum and the average expression of metal-acquisition related gene sets. Both zinc and iron concentrations in untreated sputum samples showed a significant positive correlation with the activity of metal acquisition gene sets 1, 2, 3, 4, and 6 (Fig. S6). In other words, as zinc and iron concentrations increased across the CF sputum samples, gene sets related to zinc and iron acquisition grew more, not less, active, despite the fact that treatment with metals clearly represses the activity of these gene sets. Even after FDR correction, zinc concentration remained significant (FDR < 0.01) in its association with the average expression of all five metal acquisition genes, while iron concentration did not remain significantly associated. Though surprising in the context of Fig. 4, these findings are in line with the results of prior studies where *P. aeruginosa* exhibited elevated expression of metal acquisition genes despite zinc and iron concentrations being relatively high in CF sputum (30). This phenomenon may be due to the presence in CF sputum of other factors like human calprotectin or proteins from other microbes that sequester metals (31–34). One possible explanation for the trend of increasing PAO1 metal acquisition gene set activity as metal concentrations rise is that a higher metal concentration means greater activity of these outside metal-sequestering factors, which could aggravate the *P. aeruginosa* metal restriction response.

### Type VI secretion system activity is associated with oral antibiotic and CFTR potentiator usage

In addition to the gene sets associated with metal acquisition, we identified several other gene sets that are relevant for their association with *P. aeruginosa* virulence in the CF lung. Notably, gene set 9 contains a number of genes associated with one of three type VI (T6) secretion systems (T6SS) H1-T6SS: *vgrG1c*, *tagF1*, *tssJ1*, *tssA1*, *tssC1*, *hcp1*, *tagJ1*, *tssE1*, *tssF1*, *tse5*. T6SSs enable *P. aeruginosa* to secrete effector molecules that play roles in intra- and interspecies interactions, can interface with host cells, and may contribute to the progression of lung disease in CF patients (58, 59). Notably, Hcp1 has been detected in *P. aeruginosa*-containing CF sputum and individuals with CF-associated *P. aeruginosa* infections have anti-Hcp1 antibodies (60). The constituent genes of gene set 9 are consistently more active in CF sputum compared to SCFM2 though the difference in expression was less dramatic than for the genes in the metal acquisition gene sets (Fig. S2B; Table S1). The genes in gene set 9 were not responsive to metal treatment (none of the genes in the gene set are significantly differentially expressed between the metal-treated and non-treated samples) and while higher average expression of gene set 9 was associated with oral antibiotic usage in general (*P* = 0.02, FDR = 0.20, adj. *R*^2^ = 0.32) and oral azithromycin usage in particular (*P* = 0.02, FDR = 0.51, adj. *R*^2^ = 0.29), the association is not significant after FDR correction. Interestingly, there was a stronger, significant negative association (*P* = 0.0009, FDR = 0.02, adj. *R*^2^ = 0.56) between CFTR potentiator usage and the average expression of gene set 9, indicating that CFTR potentiator treatment may reduce type VI secretion activity in *P. aeruginosa* (Fig. S5).

As an additional assessment of gene set 9 (H1-T6SS) activity, we determined how its expression across samples was related to the expression of the other gene sets as the activity of one gene set may promote or inhibit the activity of others. Alternatively, it is possible that two gene sets are responsive to the same underlying biological factors. The average expression of all identified gene sets was correlated across the *ex vivo* sputum samples (not treated with metals) using Pearson correlation. The average expression of gene set 9 across samples was correlated with the average expression of the metal acquisition gene sets (gene sets 1, 2, 3, and 4a6), especially gene sets 2 and 3 (Fig. 4A). In other words, the samples in which gene set 9 are most active are generally the same samples in which the metal acquisition gene sets are most active. Figure 4A also identifies gene sets that are anti-correlated in their average expression across samples. The metal acquisition gene sets, for example, are strongly anti-correlated in their expression across samples with gene set 8, which has associations with gluconeogenesis. Figure 4B and C demonstrate more directly the correlations between gene set 2 and gene sets 8 and 9. Notably, the three outlier samples noted earlier in the manuscript (from donors 124, 204, and 239) are distinguished by low average expression of gene sets 2 and 9, and high average expression of gene set 8.

### The *ex vivo* sputum model effectively recapitulates the transcriptome of *P. aeruginosa* clinical isolates

**Fig 5:**
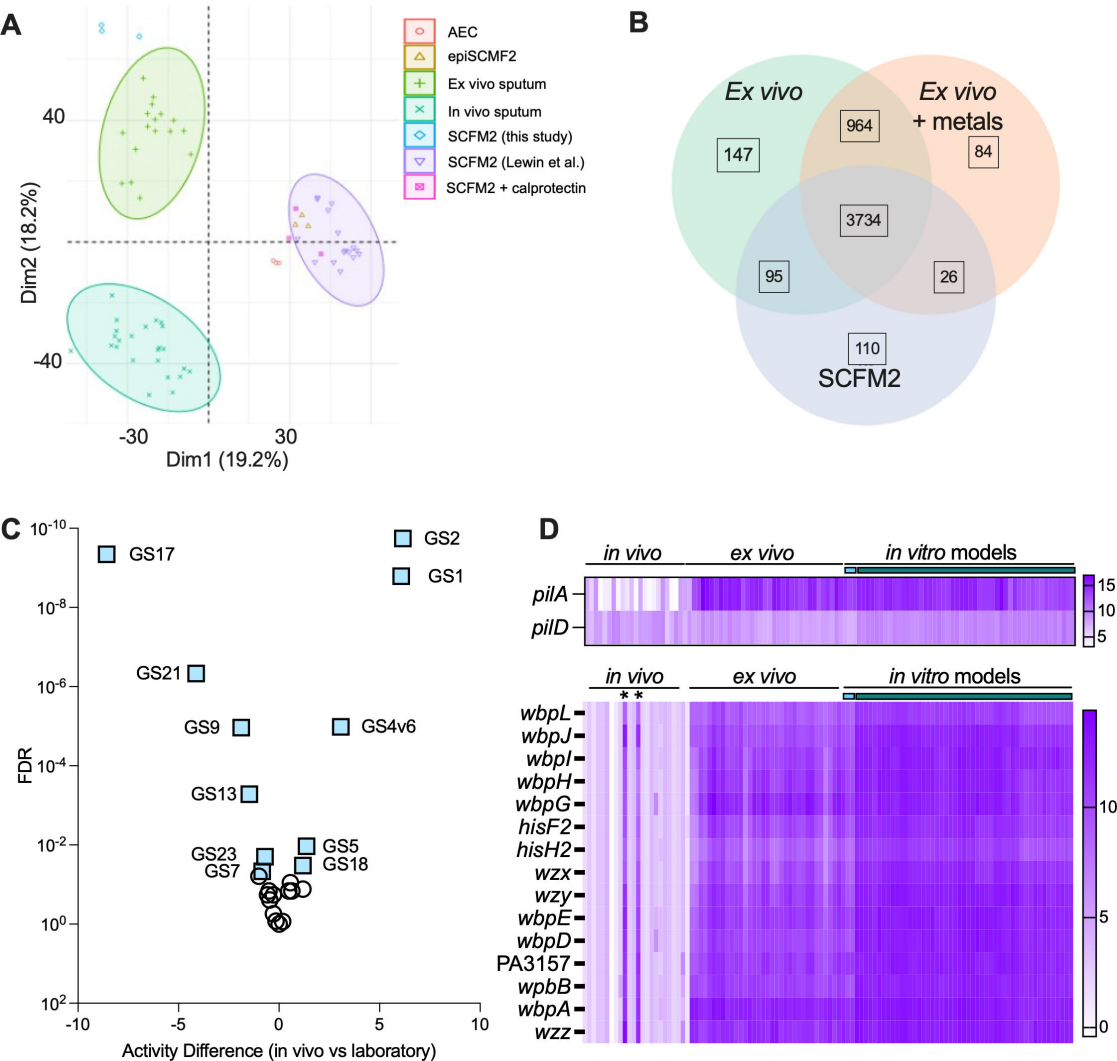
Comparison of *in vivo* and *ex vivo* sputum. (**A**) Though comparably accurate, the *ex vivo* sputum samples and the models analyzed by Lewin et al. are systematically different in their transcriptomic profiles. The *ex vivo* samples (not metal treated) and SCFM2 samples are found at the top left of the PCA plot, while the three models [SCFM2, airway epithelial cells (AEC), and epiSCFM2] from the Lewin et al. study occupy the right side of the PCA plot. The *in vivo* samples from the Lewin et al. study are the blue samples at the bottom left. Different sputum models of *P. aeruginosa* capture different features of the *in vivo P. aeruginosa* transcriptome. (**B**) The *ex vivo* model of PAO1 grown in expectorated CF sputum accurately captured 1,111 genes (147 + 964) that were not accurately captured by SCFM2. The addition of metals lowered the extent to which the *ex vivo* sputum model resembled the *in vivo* transcriptome, with 242 genes (147 + 95) accurately captured in the non-treated *ex vivo* samples that were not accurately captured in the metal-treated samples. (**C**) A volcano plot showing the difference in gene set activity (Activity Difference) and significance of the differences for each gene set (see Table 3) in a comparison to data from Lewin et al. (43) of *P. aeruginosa* RNA isolated from sputum or laboratory controls. Comparisons were made using a linear model. (**D**) Normalized counts for *pilA* (divergent across strains) and *pilD* (highly conserved across strains) or genes involved in O-antigen biosynthesis (variable by strain) in *in vivo* sputum, *ex vivo* sputum, and *in vitro* samples. The teal bar indicates SCFM2 samples from this study and the green bar indicates laboratory samples from Lewin et al. (43).

To further analyze the data we obtained using ex vivo samples, with “spiked in” *P. aeruginosa*, we compared our results to those in Lewin et al. (43) which included transcriptome analysis of endogenous *P. aeruginosa* mRNA recovered directly from expectorated sputum donated by pwCF with *P. aeruginosa* lung infections (referred to as *in vivo* transcriptomes). Notably, the *P. aeruginosa in vivo* transcriptomes used were chosen because of the high read coverage across the PAO1 genome (43), and this high coverage allowed the authors to systematically compare the *in vivo* transcriptomes to strain PAO1 transcriptomes from cells grown in several model systems—including SCFM2 with and without the metal chelating innate immune factor calprotectin, an airway epithelial cell model, and a newly-constructed combination model of SCFM2 and airway epithelial cells (epiSCFM2). The raw counts from the 37 samples from this study (17 *ex vivo,* 17 *ex vivo* + metals, and 3 SCFM2) and 63 samples from Lewin et al. (43) [24 expectorated sputum samples and 49 samples from SCFM2 without and with calprotectin, airway epithelial cells, and airway epithelial cells and ASM (epiSCFM2)] were normalized by library size. We first compared the *in vivo* transcriptomes to the *P. aeruginosa* transcriptomes from the *ex vivo* sputum samples and SCFM2-grown *P. aeruginosa* from this study and the aforementioned cell culture models using principal components analysis (Fig. 5). Along PC1, which describes ~18% of the variance, the *in vivo* and *ex vivo* samples separated from the Lewin *in vitro* models, as reported by Lewin et al. [Fig. S3 in (43)]. Among the genes found to most strongly contribute to this separation were those involved in iron uptake, sulfur metabolism, amino acid, and peptide uptake (Table S7), but no specific GO terms were enriched among these genes.

As shown in Fig. 5A, the transcriptomes from our SCFM2-grown cells did not cluster with those in (43), and we speculate that this is, at least in part, due to the differences in inoculum preparation, incubation time, and minor differences in components of the SCFM2 media recipes (43). For example, the SCFM2 cultures from this study were inoculated with cells from exponential phase cultures grown in the defined medium M63, and incubations were for 3 h in small volume cultures. The cultures in Lewin et al. (43) were inoculated from SCFM-grown cells, then grown for 16 or 16 h followed by an additional 8 h with cultured epithelial cells.

We applied the same method utilized in the Lewin et al. (43) study to assess the accuracy of transcript abundance in the *ex vivo* model relative to the clinical isolates. This involved calculating, for each gene in the Lewin et al. sputum transcriptomes, the mean transcript abundance. Then, the mean transcript abundance and z-score (number of standard deviations away from the mean of the clinical isolate samples) for each gene in the model samples was determined. If the z-score magnitude was less than two, the gene was considered accurately expressed in the model relative to the clinical isolate samples, again matching the standard in the Lewin et al. manuscript. The *ex vivo* sputum model accurately recapitulated the transcript abundance of 88.9% of genes, which compares favorably to the model systems described in the Lewin et al. study (SCFM2: 86.4%, airway epithelial cell: 84.7%, epiSCFM2: 87.8%) (Fig. 5B). By contrast, SCFM2 was 71.3% accurate, but the accuracy of SCFM2 samples for the metal-treated *ex vivo* samples was 86.4%, underscoring that SCFM2 does not model the metal availability state of *P. aeruginosa*.

Using the gene sets that we defined above, we determined if any were significantly different between the *in vivo* and *in vitro* samples from Lewin et al. (43). As shown in Fig. 5C, gene sets 1 (composed of genes induced in low zinc conditions) and gene set 2 (composed of genes responsive to iron restriction) were the most activated in the *in vivo* condition relative to the *in vitro* controls. The gene set that was most lowly expressed in *in vivo* samples was gene set 17 which contained genes that we previously found to share transcriptional patterns across strains due to low sequence identity between strains (*pilA* and *pilC*) or being strain specific such as endogenous restriction-modification systems (PA2730-2734). The last gene in gene set 17 is PA1939 which is only found in a small fraction of *P. aeruginosa* genomes. To investigate this further, we analyzed normalized expression of *pilA* (PA4525), which has striking allele differences across strains, and *pilD*, which does not show sequence divergence when different strains are compared (61) (Fig. 5D). We found that the expression of *pilA* was, on average, >200-fold higher in *ex vivo* samples than *in vivo* samples, but that some *in vivo* samples had high expression. The range of *pilA* expression across the *in vivo* samples was over 8,000-fold. In contrast, *pilD*, which does not show sequence variation, was not different between *in vivo* and *ex vivo* samples and had a fourfold range across the *in vivo* samples. As discussed further below, we predict that these differences are due to the reads from the *ex vivo* samples in this study being largely from strain PAO1 which was in excess of endogenous *P. aeruginosa*, while the *in vivo* samples contained only reads from different infecting strains of *P. aeruginosa*. Consistent with this, *pilA* was chosen as a gene to nucleate a gene set in our studies because of high expression, but not differential expression in our samples (Table 2). Other “strain specific” gene sets that were lower in *in vivo* samples than corresponding *in vitro* controls, but not different between controls and our *ex vivo* samples included the flagellar components known to show antigenic variation (gene set 21) (61).

Pathway enrichment analysis of the genes with an average expression more than fourfold higher in the *in vivo* samples compared to the *ex vivo* samples (Table S7) found one pathway: biosynthesis of alginic acid (11.5-fold enrichment with an FDR-corrected *P*-value of 2.09E−04). We speculate that the common *mucA* mutations (62) in *P. aeruginosa* CF isolates that were likely present in at least some of the *in vivo* samples led to higher levels of alginate biosynthesis genes. Genes associated with O-antigen biosynthesis were also among the most differentially expressed between the *in vivo* and *ex vivo* samples (Table S7). As for type IV pili, there were differences in mRNA levels for genes encoding the O-antigen across the *in vivo* samples relative to the *ex vivo* samples. *P. aeruginosa* has at least 20 different O-antigen types and different genes encode the different O-antigens (63). Based on expression level, it appears that human sputum samples EM30 and EM60B have O-antigen types similar to those found in strain PAO1; others likely would not be detected in data based on read mapping to the PAO1 genome. Lastly, the *in vivo* samples and control samples in (43) that differed from the *ex vivo* and control samples in this study included *mexS* which is consistent with previously described differences between PAO1 strains used in different labs (64). Together these data highlight similarities between in *in vivo* and *ex vivo* conditions and considerations that may aid in the comparison of *in vivo* and *in vitro* samples.

## DISCUSSION

The results outlined in this manuscript establish significant differences in the transcriptomic profile of *P. aeruginosa* incubated in real CF sputum compared to SCFM2. Among the transcriptional features (genes, functional terms, and correlated gene sets) most differentially active in real CF sputum are several related to metal acquisition. This finding recapitulates the results of prior studies indicating that *P. aeruginosa* experiences a metal restriction response in the CF lungs (31–34). A key takeaway of this study is that the use of SCFM2 may obscure certain aspects of *P. aeruginosa* biology that are relevant to its existence and persistence in the CF lung. To address differences between SCFM2 and CF sputum inducing the expression of particular genes, researchers may amend their medium with certain factors (for example, the introduction of calprotectin to induce a metal restriction response), choose a different variety of artificial medium (65, 66), or make use of an *ex vivo* sputum model such as that described in this study. The last approach would be ideal to recapitulate the conditions of the CF lung environment though if sputum samples from donors are not available then the other approaches would be suitable alternatives. In fact, a recent publication found that addition of calprotectin to SCFM2 (a variant of artificial sputum medium) adjusted the expression of genes to represent the *P. aeruginosa* transcriptome more accurately during active infection. This included zinc responsive genes, which were found to be relatively poorly expressed in SCFM2 (43).

The use of common laboratory strain PAO1 to identify differences in induced gene expression between SCFM2 and real CF sputum is a strength of this study in that it allows for the effects of the media to be understood directly. Differences in endogenous strains in CF sputum (e.g., the gain or loss of certain genes) may obscure the effects of the sputum environment. For example, clinical isolates from the same CF donor can have genetic differences that lead to a “substantial reprogramming of transcriptional networks” that entail reduced pyoverdine production and increased antibiotic resistance, among other changes (67). Thus, studies that explore the impact of a common *ex vivo* model on the transcriptional profile of different clinical isolates are also useful and complementary to this present study (68).

We also recommend, based on the findings of this study, that CF researchers consider patient variability when constructing and interpreting the results of laboratory models. The expression of metal restriction gene sets, and other gene sets, can vary considerably between different subgroups of patients. As a case in point, we identified three sputum samples (Spu124, Spu204, Spu239) in this study that were quite distinct in terms of the *P. aeruginosa* transcriptional profile they induced, with markedly reduced average expression of gene sets related to the tricarboxylic acid cycle (gene set 5), type-IV pilus dependent motility (gene set 17), and flagellar motility (gene set 21), among other differences. The average expression of the metal restriction gene sets was also slightly lower in these three samples than most of the other sputum samples (Fig. 3). There would be value in designing specific formulations of SCFM2—with different levels of various biological factors—that can accurately model different subgroups in the CF population. The model system discussed in this study would be useful for designing such formulations. To the system of PAO1 incubated in SCFM2, biological factors could be added in varying quantities until the gene expression profile closely resembles the profile that we observed for the different subgroups of CF sputum samples. Future studies with additional CF donors may identify new subgroups.

The approaches outlined here advance our ability to mimic CF conditions *in vivo*, but the general approach can also be used to improve our understanding of *P. aeruginosa* biology. By identifying correlated gene sets operative in CF sputum, we are striving toward a larger goal - that is, identifying external biological factors (metals, metabolites, etc.) that influence *P. aeruginosa* gene expression in different environments, characterizing the sets of genes that are influenced by these factors, and understanding the phenotypic relevance of these gene sets. In this study, we identified metal restriction gene sets that are correlated in their expression across the CF sputum samples—meaning that in certain samples the genes are more active as a whole, and in other samples, they are as a whole less active. We showed that the constituent genes are significantly better expressed in CF sputum than in SCFM2. Finally, we showed that the expression of these genes was driven by exposure to metals in a coordinated fashion: when a mixture of metals was added to the CF sputum samples, the metal restriction gene sets were repressed consistently across each sample, and all individual genes in these gene sets were significantly repressed.

Correlating gene sets with clinical parameters like FEV1 enabled us to gain insight into how host and pathogen phenotypes are intertwined. In this study there was a negative, though non-significant correlation between metal restriction gene set activity and patient FEV1. Other studies with increased power might find a stronger relationship between metal restriction gene set activity and FEV1 or may identify other gene sets whose activity is negatively associated with FEV1. Though even a strong correlation between gene set activity and FEV1 does not prove that the activity of a gene set is driving differences in clinical symptoms between patients, additional experimentation in cell culture or animal models could establish such a causal relationship between gene set activity and host phenotype. For example, researchers may manipulate the activity of a gene set, inducing or repressing the expression of its genes in a coordinated manner (as we repressed the metal restriction gene sets in this study by the addition of the metal mixture), and observe the consequences for the broader system. The challenge of this approach is that the factor used to induce or repress the gene set may also influence the host, so researchers must be careful to account for these effects. In laboratory experiments, it may be possible for *P. aeruginosa* to be pre-treated with the induction/repression factor in a separate culture before being added to cells or animal models.

Ultimately, the identification of gene sets and their driving factors may provide a new roadmap for clinical intervention. If researchers establish that a given gene set influences clinical symptoms, they can try to modulate its expression—either by targeting specific genes in the gene set or enhancing or depleting the biological factors that drive its expression. This principle can be applied to bacterial cells in the context of bacterial infection. In fact, several studies have cited the synergistic effects of combining metal chelating agents with antibiotics to improve killing of *P. aeruginosa* (69–71). The principle can also be applied to human cells—modulating the activity of correlated gene sets in individual cell types that are associated with disease symptoms. It may even apply to the microbiome. Researchers can catalog the metatranscriptomic programs operating in a coordinated fashion in the microbiome, determine their driving biological factors, and attempt to modulate their activity in a way that is beneficial for the host. It is interesting to note that researchers have compared metagenomic and metatranscriptomic data from CF sputum samples to healthy saliva samples from the Human Microbiome Project and identified various functional terms (KEGG) that are relatively active in CF sputum—including nucleotide metabolism, biosynthesis of secondary metabolites, and folate (vitamin B) biosynthesis. They also identified a relatively high abundance of siderophore transporter genes in the CF samples (72). Further identification and analysis of gene sets that are correlated in their abundance (metagenomic data) or expression (metatranscriptomic data) across samples may help identify driving factors and clinical ramifications of gene expression in the CF microbiome.

Bioinformatics tools are a major asset for advancing the research projects just outlined, specifically the development of machine-learning models that identify correlated gene sets across large collections of published samples. This study benefited greatly from the use of ADAGE, which allowed us to identify gene sets that were operative not only in our relatively small collection of CF sputum samples, but also across a wide variety of *P. aeruginosa* samples encompassing many different conditions (56, 57). The use of ADAGE gave us greater confidence that the gene sets we identified were legitimate biological programs and not an artifact of this individual study. Similar models should be developed for other pathogens, of the lung, gut, and other organs, for CF and other diseases. Furthermore, the construction of compendia that identify and characterize all gene expression data sets for pathogens relevant to CF (and other diseases) are also useful in laying the grounds for future model development (73–75).

This study does have several limitations that fellow researchers should consider. From a biological perspective, it is possible that certain nutrients which are available to *P. aeruginosa in vivo* are not present in the *ex vivo* model. Because PAO1 is spiked-in to expectorated sputum *ex vivo*, certain nutrients may have been consumed completely by the host or native microbial cells prior to spike-in and are therefore not available to PAO1. In contrast, *P. aeruginosa in vivo* may have access to these nutrients before they are fully consumed, and its transcriptional profile may look different as a consequence. Future identification of these consumed nutrients that are relevant *in vivo* would allow for the spike-in model (and other laboratory models) to be amended with these factors, which would potentially improve model accuracy.

In terms of the analysis approach, our differential gene expression analysis of PAO1 incubated in CF sputum vs SCFM2 identified many differentially expressed genes, slightly more than half of all genes detected by RNA sequencing. Differential gene expression software such as edgeR assume that most genes are not differentially expressed, and when this assumption does not hold, edgeR may identify an increased number of negative results, estimate dispersion incorrectly, and make other statistical errors. We have mitigated this issue in some respects by analyzing the gene expression data from multiple angles (differential gene expression, pathway activation analysis that does not depend on *P* value cutoffs, analysis of correlated gene sets), but further confirmation of the transcriptomic patterns observed in this study in future research would help provide additional validation. Another potential limitation is the approach we took to define correlated gene sets. We chose to define gene sets using the top 10-most correlated genes in ADAGE. While a reasonable approach, an alternative approach would be to define gene sets based on a specific edge weight cutoff in ADAGE (edge weight signifies the extent of correlation). Thus, gene sets could have a variable number of constituent correlated genes. This might identify additional correlated genes of interest.

Applications akin to ADAGE are also useful for the analysis of gene expression in human cells. HumanBase, for example, provides an atlas of functional gene networks that operate in a tissue-specific manner, built in large part by analysis of gene co-expression across public data sets in the gene expression omnibus (GEO) (76–78). This is an invaluable tool for researchers who want to understand how networks of genes are contributing to disease on the tissue or even the individual cell level. The HumanBase system is a suite of accessible bioinformatics tools maintained by the FlatIron Institute. Like ADAGE, its functional gene networks are available to researchers with any level of computational experience through a web application. This accessible approach is ideal for stimulating additional experiments conducted by wet-bench researchers.

## MATERIALS AND METHODS

### Sputum samples

Sputum samples were obtained from the DartCF Translational Research Core Specimen Bank in accordance with Dartmouth Health IRB-approved protocol 28835. Sputum had been stored in 0.5–1.5 mL aliquots at −80°C immediately after collection; the amount of time samples was stored frozen varied. Each sample was thawed once, homogenized through an 18 gauge needle, and stored in 100 µL aliquots at −80°C until *ex vivo* transcriptome analyses were performed.

### *P. aeruginosa* transcriptome analysis in *ex vivo* sputum

*P. aeruginosa* PAO1 (DH294) was grown overnight in 5 mL LB (lysogeny broth) on a roller drum at 37°C for 16 h (79). From this culture, 1 mL was used to inoculate 50 mL M63 0.2% glucose amended with metals (3 µM ammonium ferrous sulfate, 1.5 µM zinc sulfate, and 0.1 µM manganese chloride). The metal concentrations were chosen empirically so that they did not suppress siderophore and zincophore-encoding gene expression and also fell within an order of magnitude of the lowest observed respective Fe^2+^, Zn^2+_,_^ and Mn^2+^ concentrations observed in the sputum samples (see Table S6 of metal concentrations). Cells were spun down and washed twice in dH_2_0 and re-suspended in 500 µL dH_2_0. Ten microliters of the cell suspension was added to 100 µL aliquots of (i) sputum, (ii) sputum amended with 10 µL dH_2_0 or metals solution (300 µM ammonium ferrous sulfate, 150 µM zinc sulfate, and 10 µM manganese chloride)—these metal concentrations were also chosen empirically to suppress siderophore and zincophore-encoding gene expression and fall within an order of magnitude of the highest observed respective Fe^2+^, Zn^2+^, and Mn^2+^ concentrations observed in the sputum samples (see Table S6 of metal concentrations), (iii) SCFM2 (42), or (iv) M63* in 1.5 mL Eppendorf tubes. Tubes were taped on their side with the lids open, placed in a humidity chamber, and incubated at 37°C with shaking at 250 RPM for 3 h. Sputum was not sterilized prior to spike-in and total RNA, from both previously-existing and spiked-in organisms, was extracted using the Zymo Direct-zol RNA extraction kit. On-column DNase I treatment (cat# R2061) and small RNA fragments (including degraded mRNA) were separated into a separate fraction using the Zymo RNA Clean and Concentrator kit (cat# R1017). The comparator cultures in SCFM2 or M63 were similarly incubated. This manuscript outlines the gene expression differences between PAO1 incubated in SCFM2 compared to sputum (metal-untreated or metal-treated). Though not emphasized in the manuscript, the aforementioned M63 samples are included in the raw RNA-seq count data, accessible through the Github repository associated with this publication, and available for re-analysis by future researchers who want to evaluate differences in *P. aeruginosa* gene expression when incubated in M63 and the other media types investigated in this study.

### RNA sequencing

RNA-seq was performed through the Microbial Genome Sequencing Center (MIGS) and processed via a Salmon v1.5.2-based pipeline on the Dartmouth College HPC cluster. For all samples, the percentage of reads that mapped to *P. aeruginosa* (vs human and other microbial species) was determined and is shown in Fig. S7. Specifically, reads were mapped to the PAO1 reference genome. Although the amount of PAO1 spiked into the sputum samples was relatively high, a small proportion of the PAO1-mapping reads may have derived from native isolates in the expectorated sputum that are genetically similar to PAO1. Even so, the normalization of raw count data as described in the results ensured that any differences in library size between samples due to varying amounts of native isolates in the samples were accounted for. Furthermore, whether a PAO1-mapping read came from PAO1 itself or a native isolate, the essential question of what genes are expressed in sputum is still answered.

### Differential gene expression and pathway analysis

To identify genes that are differentially expressed in CF spike-in sputum samples exposed to metal treatment vs samples not exposed to metals, the R package edgeR was used (v 3.40.2) (46). The edgeR package was also used to identify differentially expressed genes in the spike-in samples compared to SCFM2. In both cases, the ESKAPE Act PLUS web application was used to determine significantly activated or repressed GO terms associated with the differentially expressed genes (47). This activation analysis involves a binomial test, where the significance of pathway activation is dependent on the number of genes in the pathway with a positive (or negative) fold change, not on the magnitude of the fold change or *P* value calculated by edgeR. The second section of the results provides further rationale for the use of this approach. The ESKAPE Act PLUS web application is available at the following link: http://scangeo.dartmouth.edu/ESKAPE/. Pathway enrichment analysis for genes differentially expressed between *in vivo* genes and controls was performed at geneontology.org/docs/go-enrichment-analysis.

### Key gene identification and gene set construction

Gene sets were constructed as outlined in the results. First, genes that were highly abundant and differentially expressed in the spike-in sputum samples compared to SCFM2, or highly abundant in their expression across the spike-in sputum samples, were determined. The most differentially active genes in CF sputum relative to SCFM2 were determined by filtering the differential gene expression results (PAO1 in CF sputum vs SCFM2) for genes with positive fold change, then selecting those with fold change and logCPM values both in the top 10%, then filtering further for genes with an FDR-corrected *P*-value less than 0.05. Fold change references the extent of differential expression between two experimental conditions, while logCPM is a measure of how abundantly expressed a gene is on average across samples. The genes in the second category constitute the top 50 most abundantly expressed genes on average across all the spike-in sputum samples, after normalizing gene expression counts across samples by library size. The identification of key genes that meet these different criteria are outlined in Table S2.

Ultimately, genes in both categories were consolidated (12 genes were included in both categories—PA2402, PA3126, PA2424, PA2399, PA5556, PA2400, PA4263, PA4568, PA4432, PA4470, PA4741, PA4262—and there were 35 unique key genes in total presented in Table 2). The list of key genes presented in Table 2 excludes basic maintenance genes, which were identified by their associated GO biological process terms. Specifically, we removed genes solely associated with any of the following GO BP terms related to transcription [DNA-templated transcription (GO:0006351), DNA-templated transcription initiation (GO:0006352), regulation of DNA-templated transcription (GO:0006355)], translation [translation (GO:0006412), translational elongation (GO:0006414), cytoplasmic translation (GO:0002181), negative regulation of translation (GO:0017148), tRNA N1-guanine methylation (GO:0002939), ribosomal small subunit biogenesis (GO:0042274), ribosomal large subunit assembly (GO:0000027), rRNA processing (GO:0006364), ribosome biogenesis (GO:0042254), ribosome disassembly (GO:0032790)], ATP synthesis [proton motive force-driven ATP synthesis (GO:0015986), proton motive force-driven plasma membrane ATP synthesis (GO:0042777)], and cell division [cell cycle (GO:0007049), cell division (GO:0051301), negative regulation of cell division (GO:0051782)]

From each of these key genes, the web tool ADAGE (56) was used to construct sets of 10 genes in total (including the key gene) that are highly correlated in their expression with the key genes across the compendium of *P. aeruginosa* samples that ADAGE was based on. ADAGE is available at the following link: https://adage.greenelab.com/.

Subsequently, the constructed gene sets were consolidated—gene sets with five or more of the same genes were merged—then pruned, so that they only contained genes which were well correlated in their expression across the spike-in sputum samples. For a constituent gene to be considered well correlated enough to remain in the gene set, its Pearson correlation coefficient (*r*) with the key gene had to be greater than 0.5. The final list of consolidated, pruned gene sets is what appears in Table 3. The several steps of consolidating and pruning gene sets are recorded in Table S2.

As a final measure to strengthen our confidence that the gene sets are broadly relevant biological signatures, they were further checked for internal correlation across an independent compendium of 890 PAO1 gene expression samples (80). These samples were processed as described in the associated publications (44, 80). For every one of the 24 gene sets in Table 3, all constituent genes remained positively correlated (Pearson correlation coefficient > 0) in the independent compendium (Fig. S8).

### Linear regression analysis

To determine whether there were any significant associations between the average expression of gene sets and the metadata gathered for the donors, including metal concentrations in sputum, linear regression was performed in R using the built-in linear model function “lm().” For each combination of gene set and clinical parameter, a linear regression model was constructed (e.g., *Y* ~ *X*, where *Y* = average gene set expression and *X* = potentiator usage yes/no). *P* values and *R*² values are based on these simple models. Because we tested for the significant association of the 24 different gene sets with each clinical parameter, the *p*.adjust function was used to adjust any *P* values reported in the manuscript (method = FDR, *n* = 24 *P* values).

### Analysis code and figure production

All code was created using the RStudio integrated development environment (IDE) (81). The package edgeR was used throughout the manuscript for differential gene expression analysis (82). A number of additional outside packages were used to generate figures for the manuscript. The gplots package (v 3.1.3) was used to generate the heat map in Fig. 3A (83). The ggplot2 package (v 3.4.0) and ggpubr package (v 0.5.0) were used to generate the paired boxplots in Fig. 4 and Fig. S2, with a theme from the ggprism (v 1.0.4) package also used to shape figure appearance (84–86). The PCA plots in Fig. 1B and 2A were created with the packages factoextra (v 1.0.7) and FactoMineR (v 2.6) (87, 88). The Venn Diagram in Fig. 1A was created with the Venn Diagram (v1.7.3) package (89). The R package dplyr (v 1.0.10) was used to structure data and facilitate analysis (90). The heatmaps in figure panels 2C, 2D, 3B, and 3C were created using the R packages ComplexHeatmap (v2.14.0) and circlize (v0.4.15) (91–93). The corrplot package (v 0.92) was used to produce figure panel 5A (94). The analysis code and data inputs are provided in the associated Github Repository, which was archived in Zenodo prior to submission: https://zenodo.org/badge/latestdoi/670576663

## Data Availability

The RNA-seq count data and metadata described in this manuscript and the analysis code are both accessible at the following Github Repository. The raw RNA sequencing data have been uploaded to NCBI and are available at accession no. PRJNA1006084.

## SUPPLEMENTAL MATERIAL

Supplemental figures spectrum.03157-23-s0001.pdf Fig. S1 to S7.

Table S1 spectrum.03157-23-s0002.xlsx Differential gene expression analysis: spike-in CF sputum samples vs. SCFM2 samples.

Table S2 spectrum.03157-23-s0003.xlsx Identification of key genes that define the PAO1 transcriptional profile in CF sputum and construction of correlated gene sets in three stages (initial gene sets, consolidated gene sets, and final, pruned gene sets).

Table S3 spectrum.03157-23-s0004.xlsx Average expression of all correlated gene sets across every sample.

Table S4 spectrum.03157-23-s0005.xlsx Median values for average gene set expression compared between CF sputum and ASM.

Table S5 spectrum.03157-23-s0006.xlsx Differential gene expression analysis: spike-in CF sputum samples, metal treated vs. untreated.

Table S6 spectrum.03157-23-s0007.xlsx Metal analysis of donated *ex vivo* sputum.

Table S7 spectrum.03157-23-s0008.xlsx Normalized RNA Seq data for *ex vivo* and *in vivo* samples and their *in vitro* comparators.

Table S8 spectrum.03157-23-s0009.xlsx PCA of *in vivo* and *ex vivo* samples.

ASM does not own the copyrights to Supplemental Material that may be linked to, or accessed through, an article. The authors have granted ASM a non-exclusive, world-wide license to publish the Supplemental Material files. Please contact the corresponding author directly for reuse.
