# Supplementary material for: Pseudomonas aeruginosa transcriptome analysis of metal restriction in ex vivo cystic fibrosis sputum
Source: Microbiol Spectr. 2024 Feb 22;12(4):e03157-23. doi: 10.1128/spectrum.03157-23 (PMC10986534; doi:10.1128/spectrum.03157-23)
Supplement: Supplemental figures — Fig. S1 to S7. [file spectrum.03157-23-s0001.pdf]

Supplementary Figures

Figure S1. Heat maps demonstrating the expression of constituent genes in the 23 gene sets across the spike-in sputum (untreated and metal-treated) and ASM samples. In these panels, darker red boxes represent relatively higher gene expression, while lighter yellow boxes represent relatively lower expression. The heat maps compare metal-treated sputum samples to untreated sputum samples, and also include ASM samples.

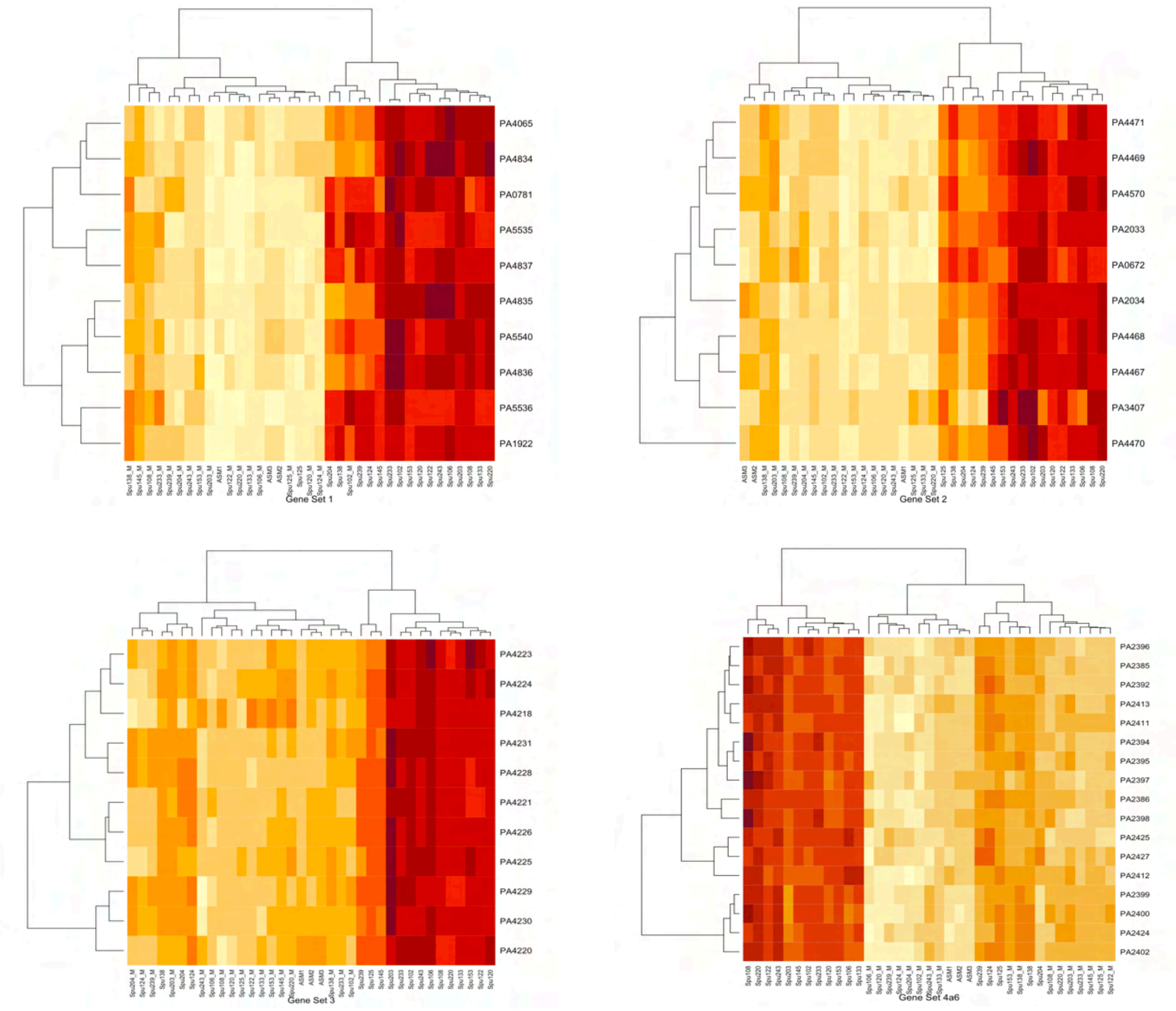

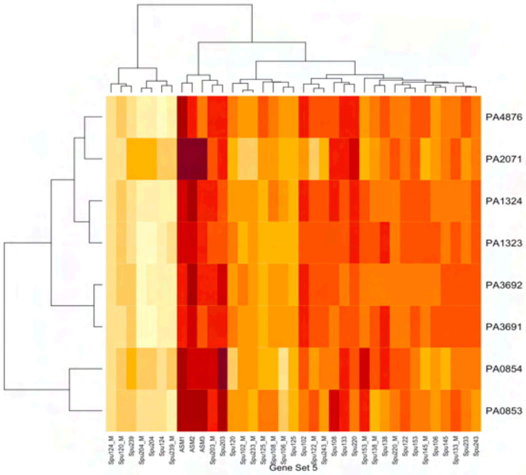
PA4876
PA2071
PA1324
PA1323
PA3692
PA3691
PA0854
PA0853
Gene Set 5

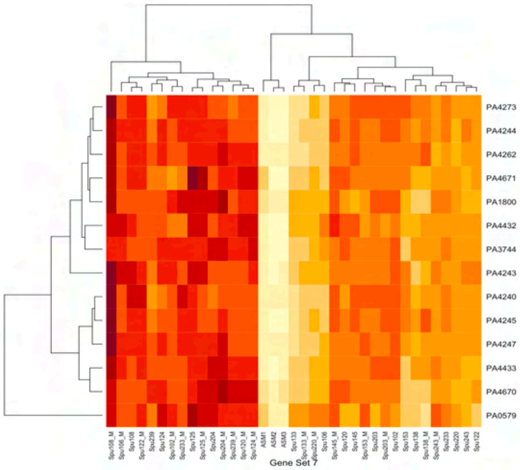
PA4273
PA4244
PA4262
PA4671
PA1800
PA4432
PA3744
PA4243
PA4240
PA4245
PA4247
PA4433
PA4670
PA0579
Gene Set 7

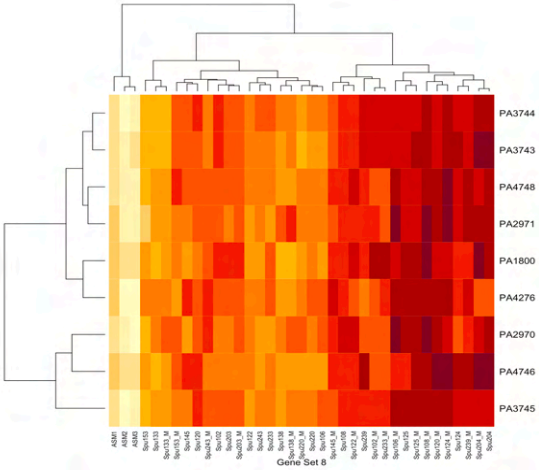
PA3744
PA3743
PA4748
PA2971
PA1800
PA4276
PA2970
PA4746
PA3745
Gene Set 8

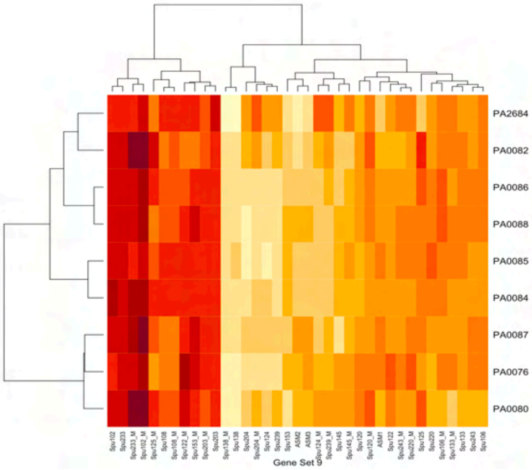
PA2684
PA0082
PA0086
PA0088
PA0085
PA0084
PA0087
PA0076
PA0080
Gene Set 9

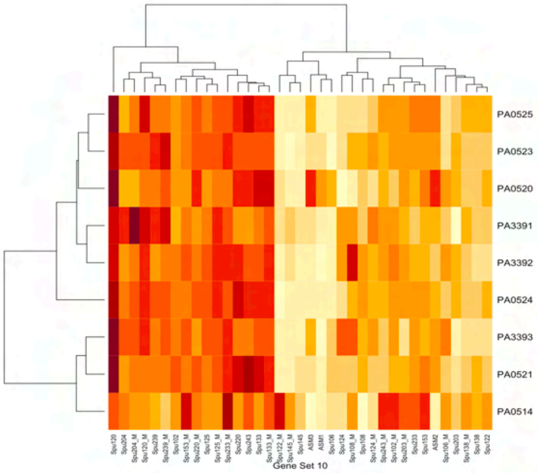
PA0525
PA0523
PA0520
PA3391
PA3392
PA0524
PA3393
PA0521
PA0514
Gene Set 10

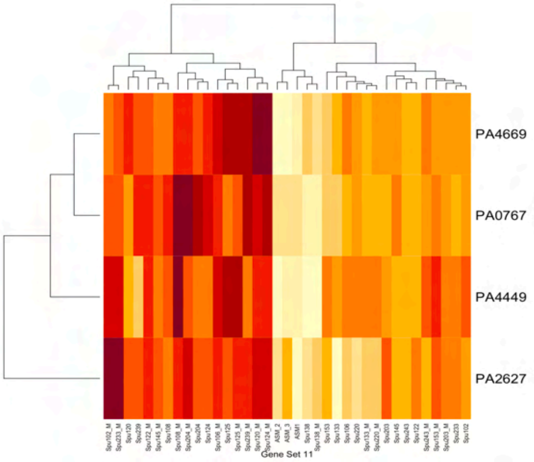
PA4669
PA0767
PA4449
PA2627
Gene Set 11

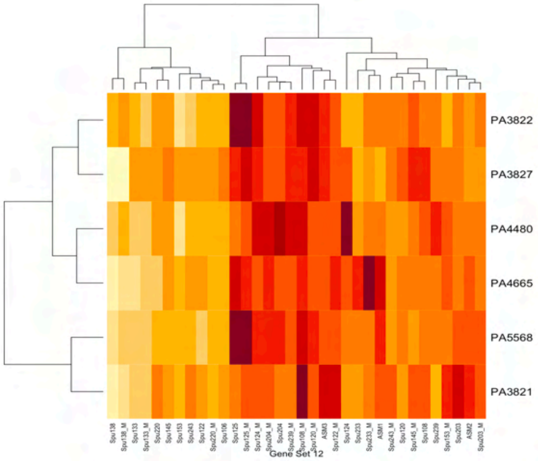
PA3822
PA3827
PA4480
PA4665
PA5568
PA3821
Gene Set 12

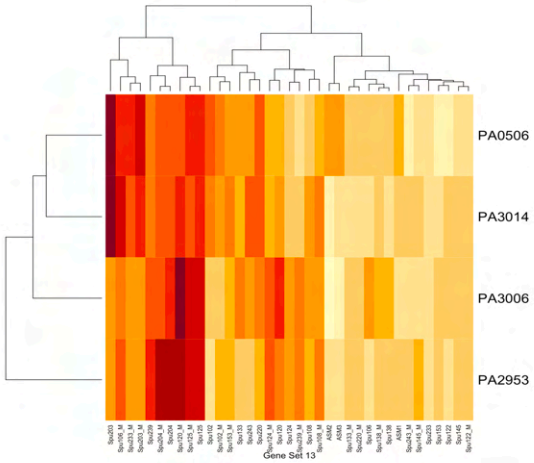
PA0506
PA3014
PA3006
PA2953
Gene Set 13

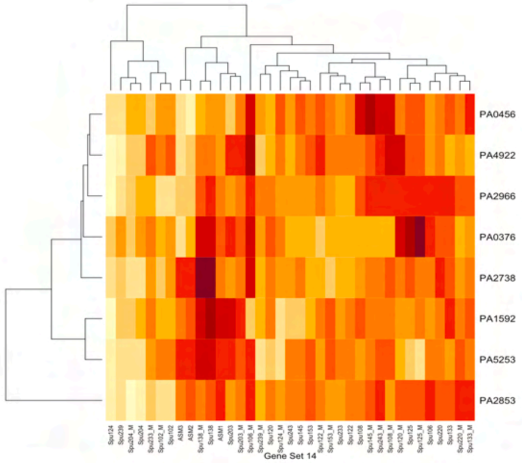
PA0456
PA4922
PA2966
PA0376
PA2738
PA1592
PA5253
PA2853
Gene Set 14

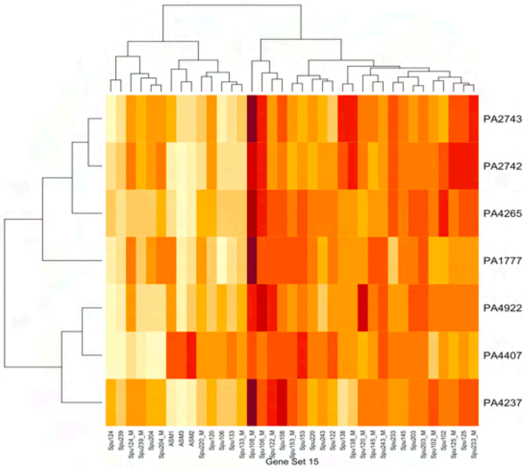
PA2743
PA2742
PA4265
PA1777
PA4922
PA4407
PA4237
Gene Set 15

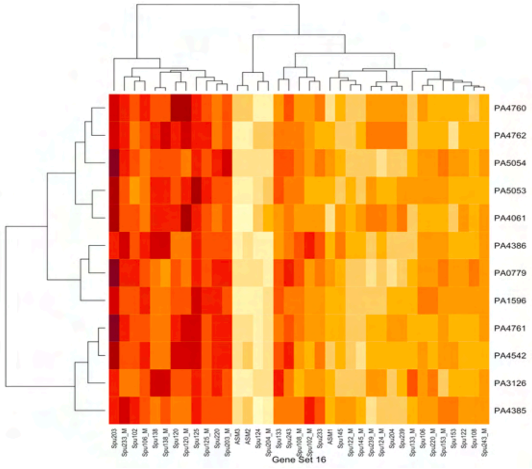
PA4760
PA4762
PA5054
PA5053
PA4061
PA4386
PA0779
PA1596
PA4761
PA4542
PA3126
PA4385
Gene Set 16

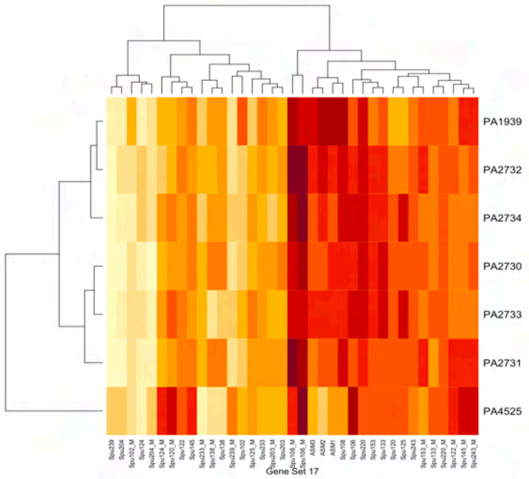
PA1939
PA2732
PA2734
PA2730
PA2733
PA2731
PA4525
Gene Set 17

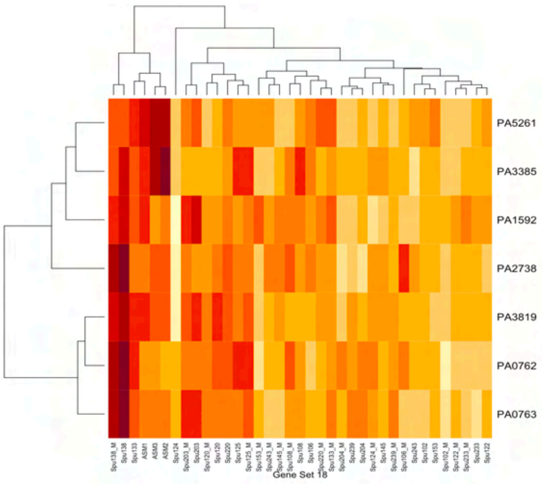
PA5261
PA3385
PA1592
PA2738
PA3819
PA0762
PA0763
Gene Set 18

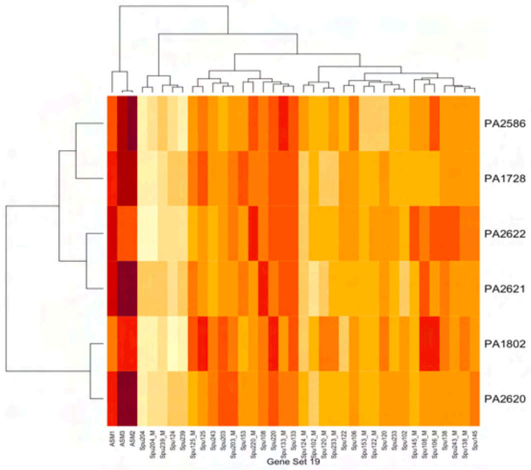
PA2586
PA1728
PA2622
PA2621
PA1802
PA2620
Gene Set 19

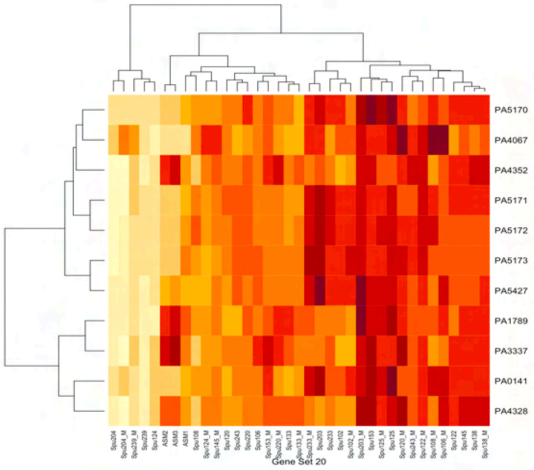
PA5170
PA4067
PA4352
PA5171
PA5172
PA5173
PA5427
PA1789
PA3337
PA0141
PA4328
Gene Set 20

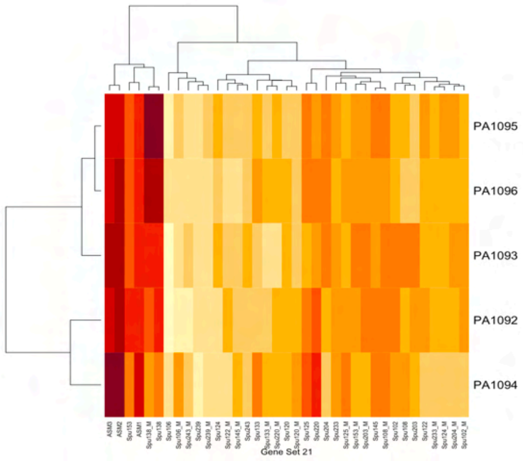
PA1095
PA1096
PA1093
PA1092
PA1094
Gene Set 21

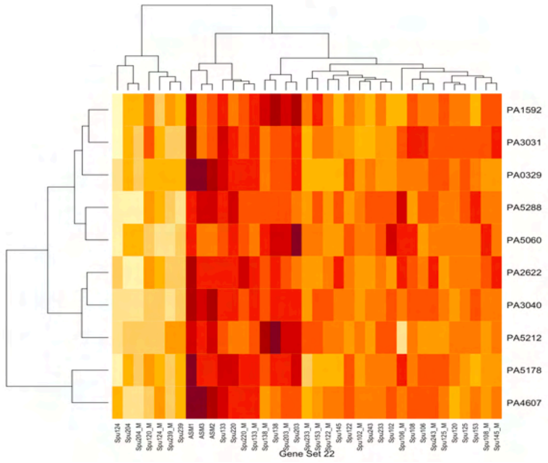
PA1592
PA3031
PA0329
PA5288
PA5060
PA2622
PA3040
PA5212
PA5178
PA4607
Gene Set 22

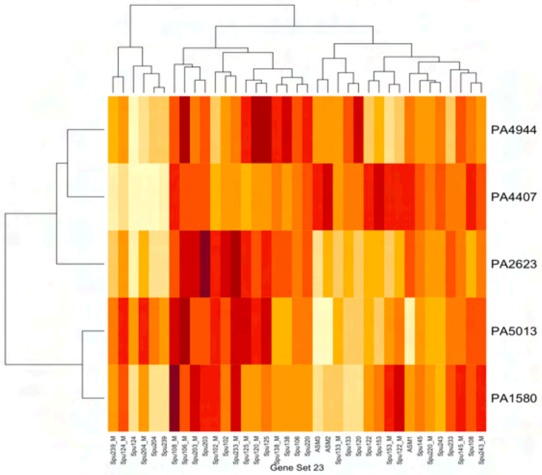
PA4944
PA4407
PA2623
PA5013
PA1580
Gene Set 23

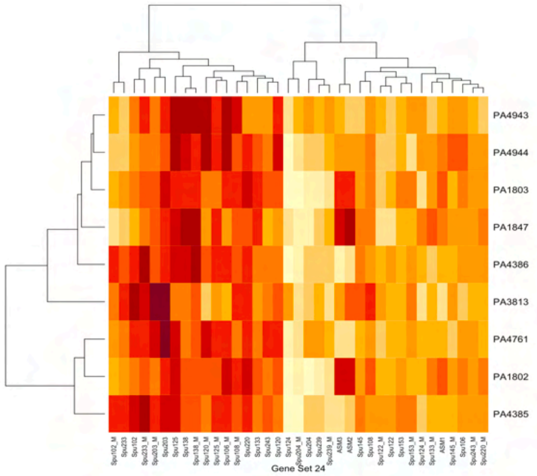
PA4943
PA4944
PA1803
PA1847
PA4386
PA3813
PA4761
PA1802
PA4385
Gene Set 24

Figure S2. Response to metal exposure for all 23 ADAGE-constructed gene sets [Table S2]. The activity score was calculated for each gene set on a sample-by sample basis. (A) The box plots represent the activity scores of CF sputum sample not treated with metals (blue boxes) and the corresponding metal-treated samples (yellow boxes). The red line indicates the median activity score for each gene set across the ASM samples. (B) The corresponding volcano plots show the differential expression of genes between spike-in sputum (untreated) and ASM samples.

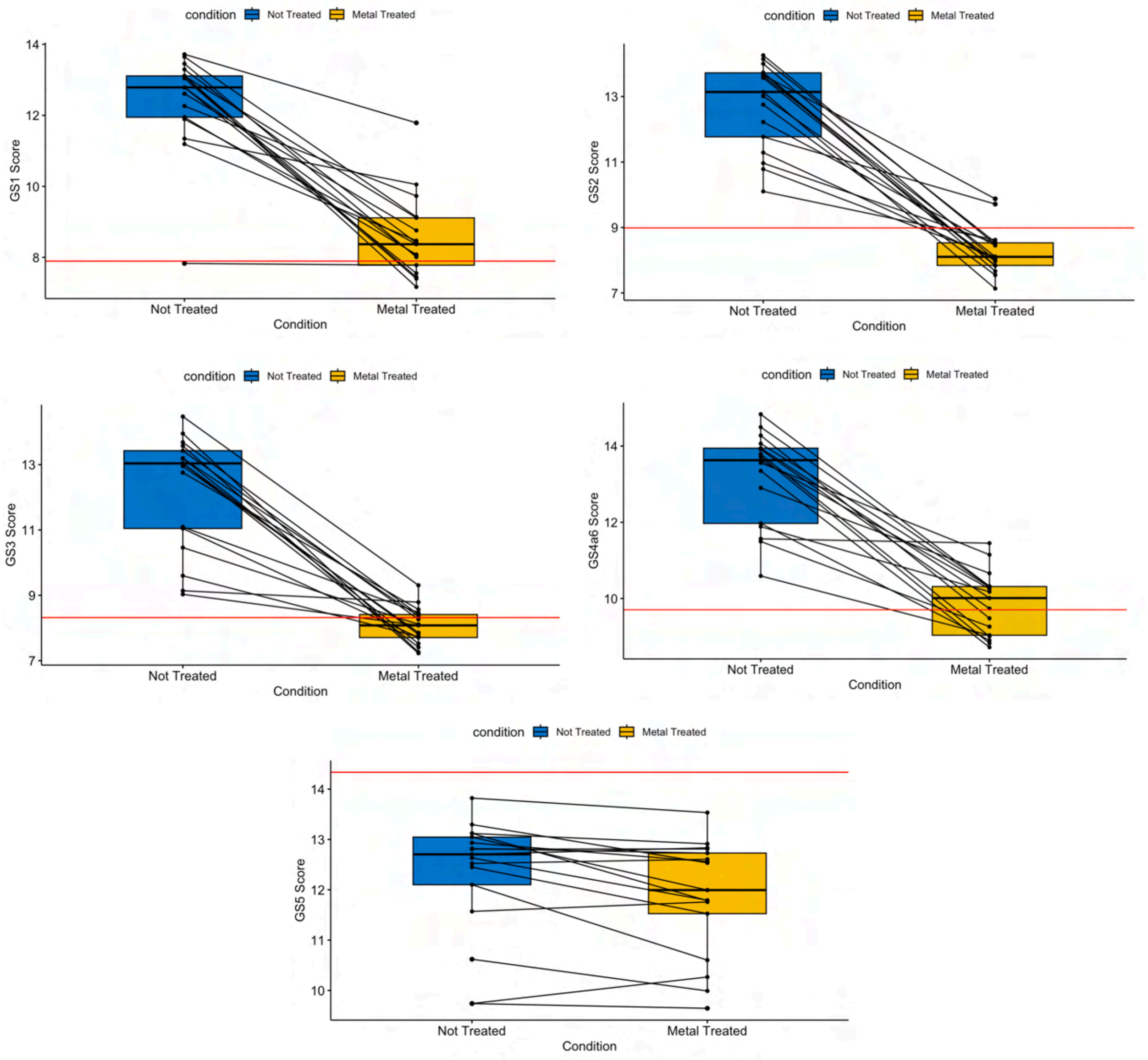

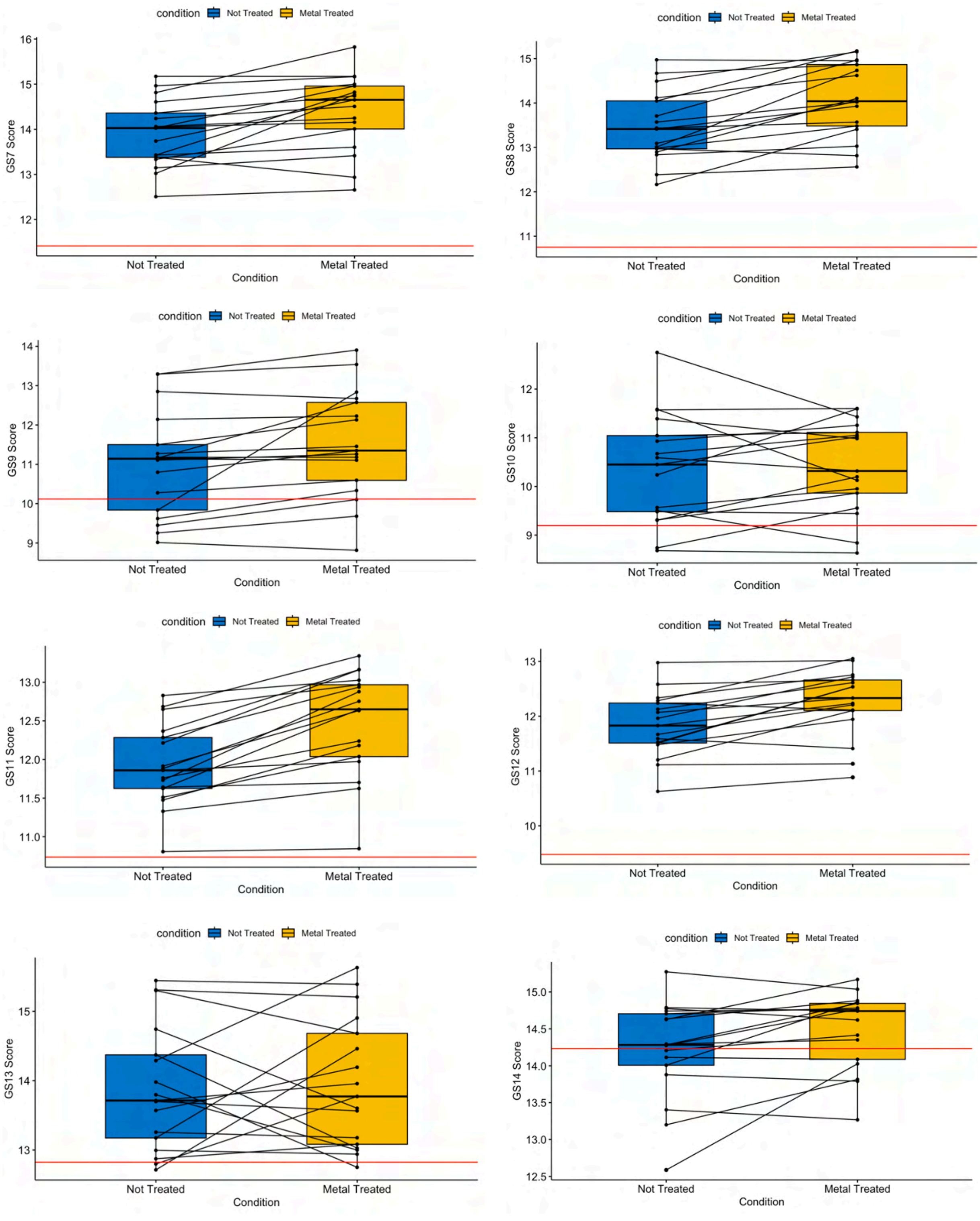
condition
Not Treated
Metal Treated
GS7 Score
GS8 Score
GS9 Score
GS10 Score
GS11 Score
GS12 Score
GS13 Score
GS14 Score
Not Treated
Metal Treated
Condition

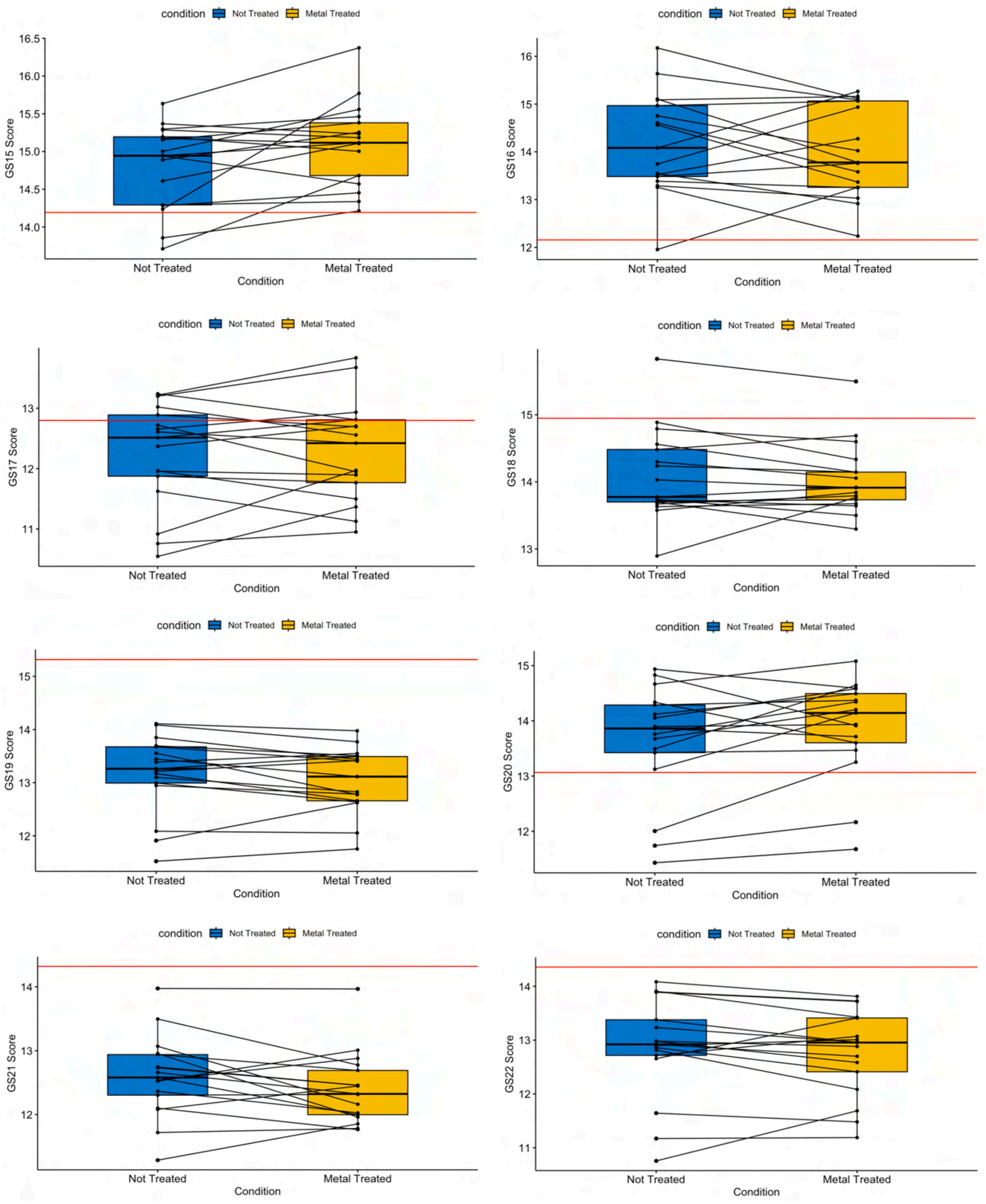
condition
Not Treated
Metal Treated
GS15 Score
GS16 Score
GS17 Score
GS18 Score
GS19 Score
GS20 Score
GS21 Score
GS22 Score
Condition

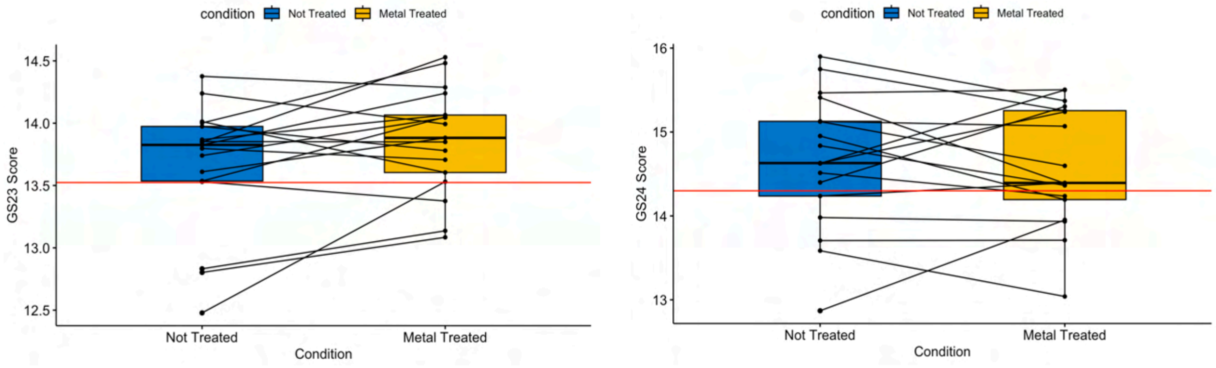

Figure S2B. Volcano plots demonstrating differential expression of genes in the spike-in sputum samples (untreated) compared to ASM. Genes within the indicated gene set are in red.

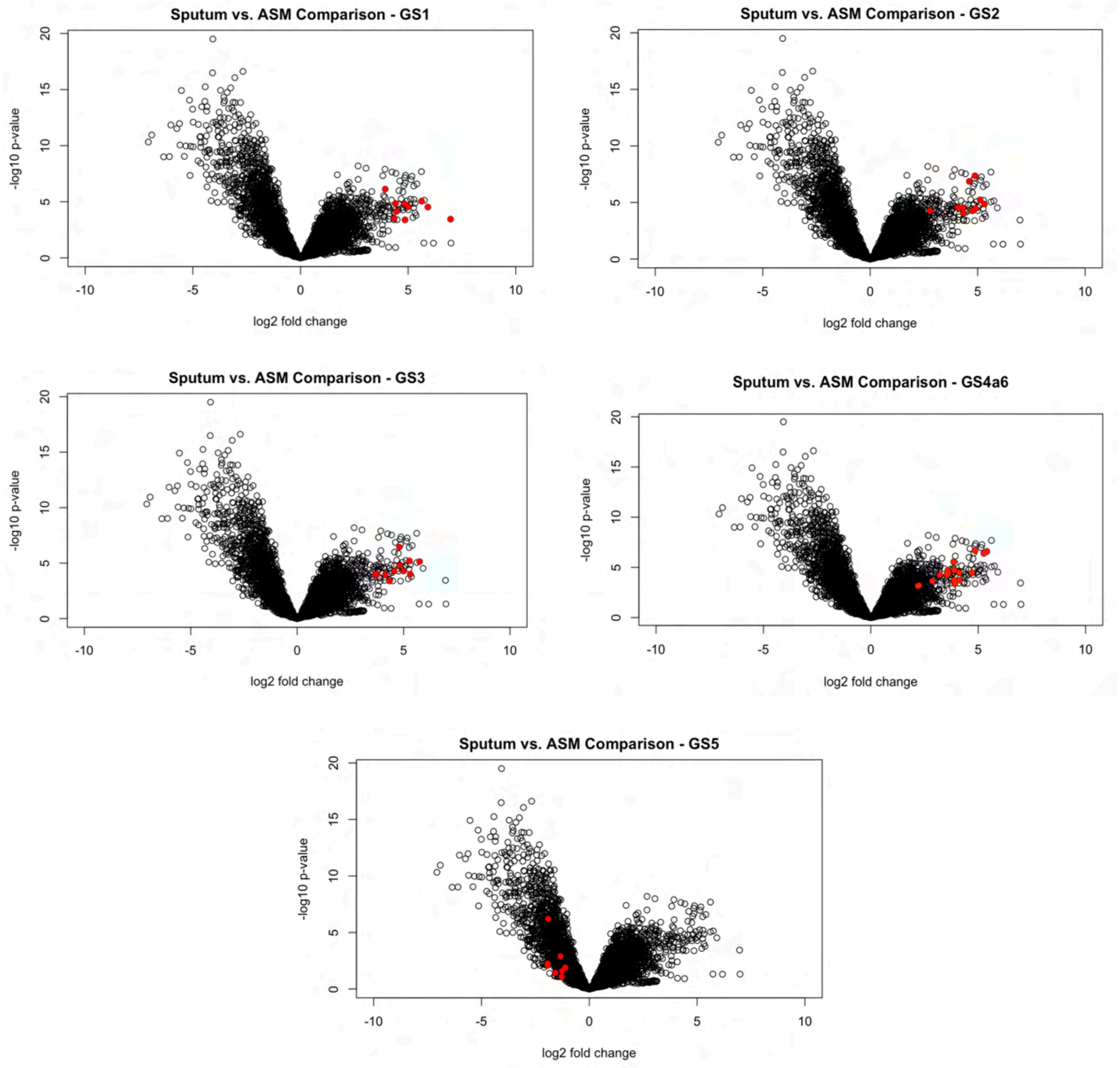

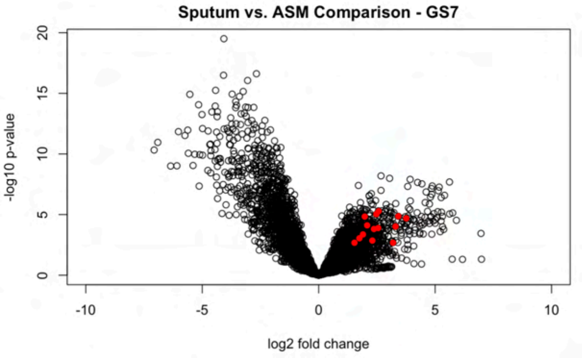
Sputum vs. ASM Comparison - GS7
-log10 p-value
log2 fold change

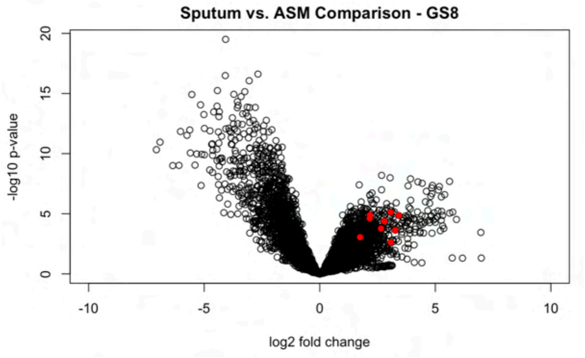
Sputum vs. ASM Comparison - GS8
-log10 p-value
log2 fold change

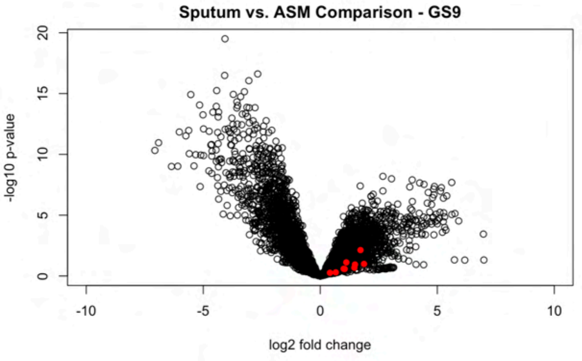
Sputum vs. ASM Comparison - GS9
-log10 p-value
log2 fold change

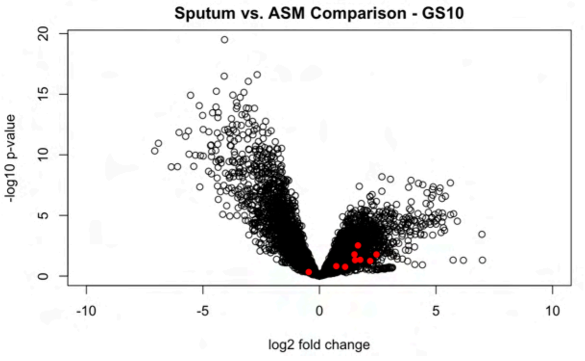
Sputum vs. ASM Comparison - GS10
-log10 p-value
log2 fold change

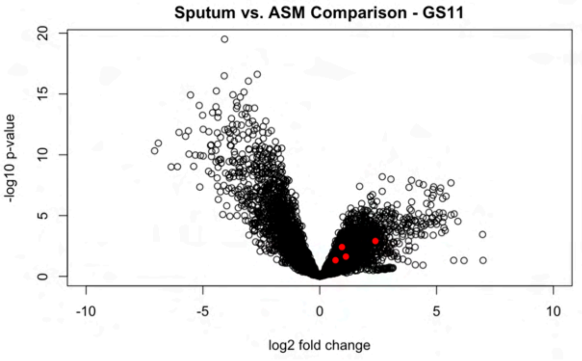
Sputum vs. ASM Comparison - GS11
-log10 p-value
log2 fold change

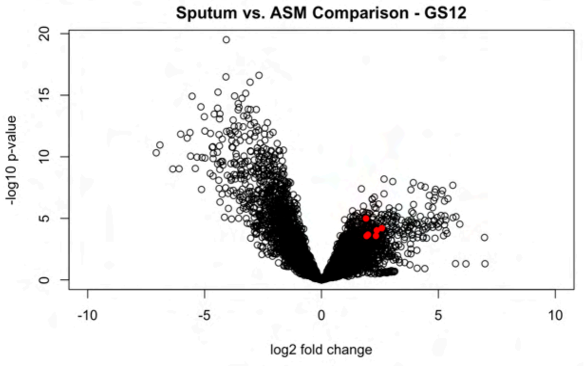
Sputum vs. ASM Comparison - GS12
-log10 p-value
log2 fold change

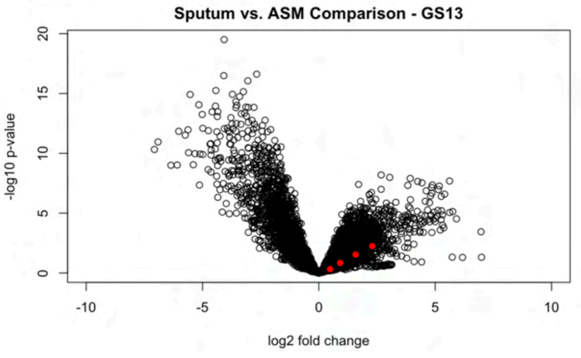
Sputum vs. ASM Comparison - GS13
-log10 p-value
log2 fold change

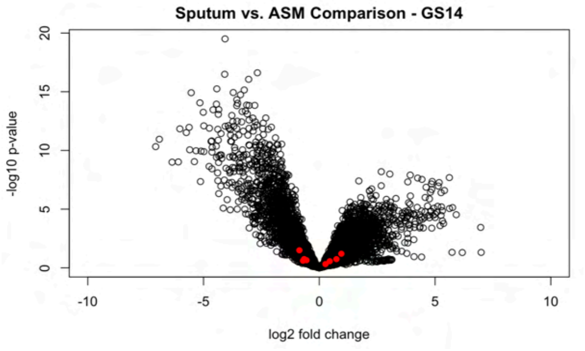
Sputum vs. ASM Comparison - GS14
-log10 p-value
log2 fold change

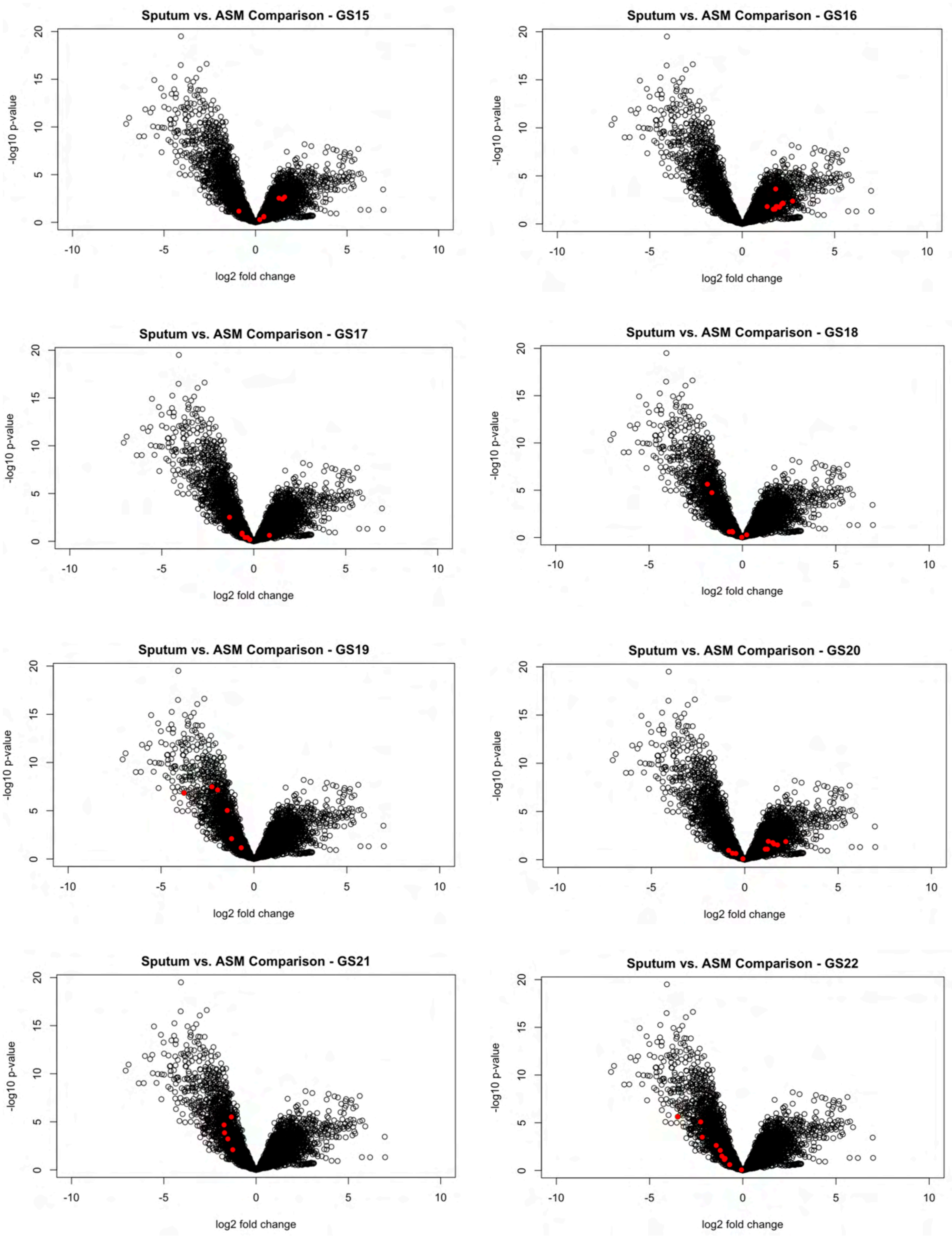

Sputum vs. ASM Comparison - GS15
Sputum vs. ASM Comparison - GS16
Sputum vs. ASM Comparison - GS17
Sputum vs. ASM Comparison - GS18
Sputum vs. ASM Comparison - GS19
Sputum vs. ASM Comparison - GS20
Sputum vs. ASM Comparison - GS21
Sputum vs. ASM Comparison - GS22
-log10 p-value
log2 fold change

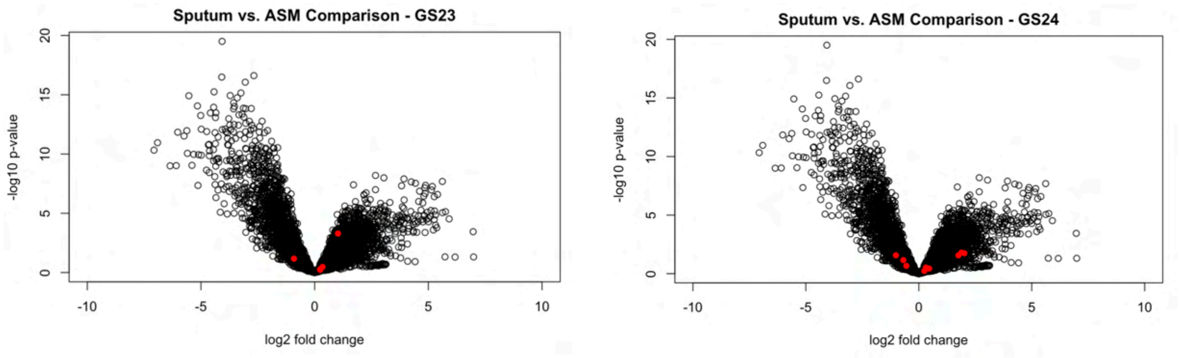

Figure S3. GO terms that are significantly activated or repressed by the addition of the metal mixture to the spike-in sputum samples. This comparison involves just the 17 untreated spike-in samples and the 17 corresponding metal-treated samples. As noted in the manuscript, each donor has a corresponding treated and untreated sample, as noted in the manuscript.

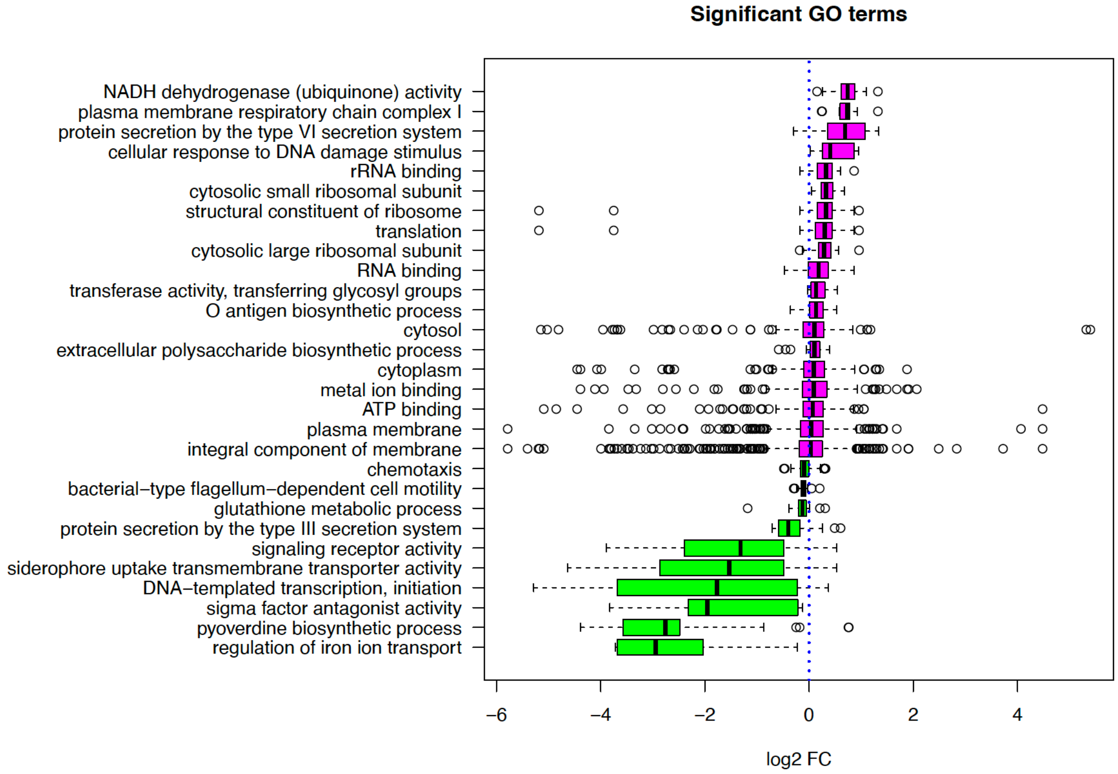

Figure S4. Linear regression analysis demonstrates the relationship between donor FEV1 and the average expression of metal acquisition gene sets.

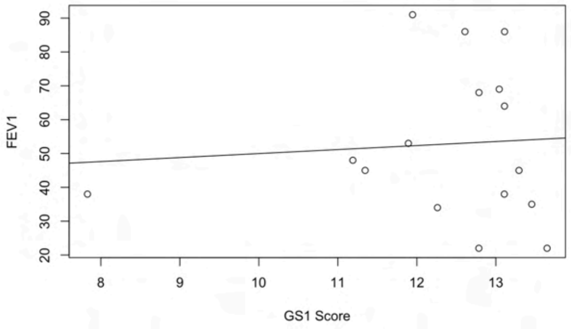


```
Call:
lm(formula = Donor_Activation_DF_1$FEV1 ~ Donor_Activation_DF_1$GS1_Score)

Residuals:
    Min      1Q  Median      3Q     Max
-32.300 -16.410  -4.983  14.894  38.714

Coefficients:
                                Estimate Std. Error t value Pr(>|t|)
(Intercept)                       38.149     52.418   0.728    0.479
Donor_Activation_DF_1$GS1_Score    1.183      4.223   0.280    0.783

Residual standard error: 22.98 on 14 degrees of freedom
Multiple R-squared:  0.005578,  Adjusted R-squared:  -0.06545
F-statistic: 0.07853 on 1 and 14 DF,  p-value: 0.7834
```

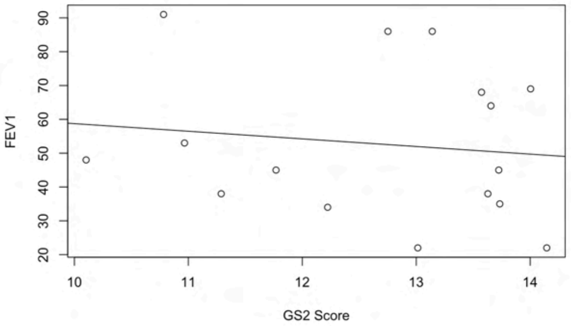


```
Call:
lm(formula = Donor_Activation_DF_1$FEV1 ~ Donor_Activation_DF_1$GS2_Score)

Residuals:
    Min      1Q  Median      3Q     Max
-29.944 -15.950  -7.546  17.811  34.342

Coefficients:
                                Estimate Std. Error t value Pr(>|t|)
(Intercept)                       81.299     57.848   1.405    0.182
Donor_Activation_DF_1$GS2_Score   -2.256      4.548  -0.496    0.628

Residual standard error: 22.84 on 14 degrees of freedom
Multiple R-squared:  0.01727,   Adjusted R-squared:  -0.05293
F-statistic: 0.246 on 1 and 14 DF,  p-value: 0.6276
```

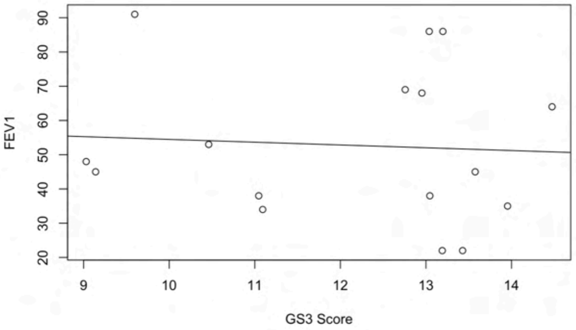


```
Call:
lm(formula = Donor_Activation_DF_1$FEV1 ~ Donor_Activation_DF_1$GS3_Score)

Residuals:
    Min      1Q  Median      3Q     Max
-29.882 -15.786  -6.918  16.135  36.194

Coefficients:
                                Estimate Std. Error t value Pr(>|t|)
(Intercept)                       62.619     40.816   1.534    0.147
Donor_Activation_DF_1$GS3_Score   -0.814      3.333  -0.244    0.811

Residual standard error: 22.99 on 14 degrees of freedom
Multiple R-squared:  0.004242,  Adjusted R-squared:  -0.06688
F-statistic: 0.05964 on 1 and 14 DF,  p-value: 0.8106
```

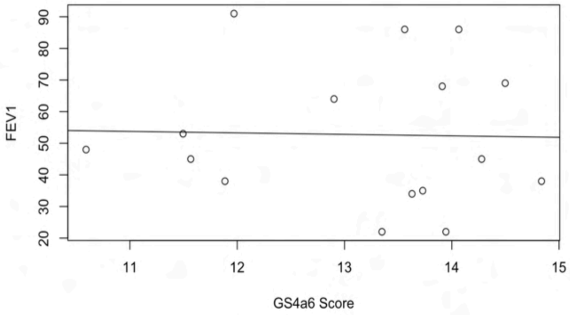


```
Call:
lm(formula = Donor_Activation_DF_1$FEV1 ~ Donor_Activation_DF_1$GS4a6_Score)

Residuals:
    Min      1Q  Median      3Q     Max
-30.651 -15.873  -6.581  15.933  37.700

Coefficients:
                                  Estimate Std. Error t value Pr(>|t|)
(Intercept)                        58.9398    62.5687   0.942    0.362
Donor_Activation_DF_1$GS4a6_Score  -0.4711     4.7420  -0.099    0.922

Residual standard error: 23.03 on 14 degrees of freedom
Multiple R-squared:  0.0007045, Adjusted R-squared:  -0.07067
F-statistic: 0.00987 on 1 and 14 DF,  p-value: 0.9223
```

Figure S5. Linear regression analysis demonstrates the association between drug usage and average expression of gene set 9 (type VI secretion) as well as metal acquisition gene sets.

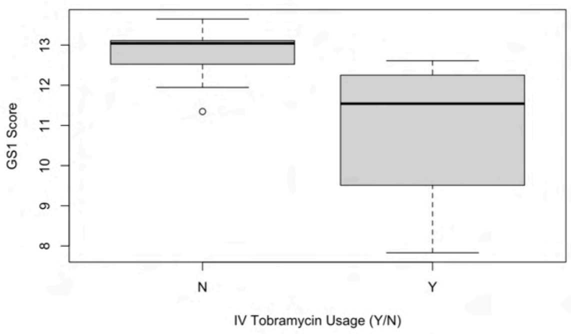


```
Call:
lm(formula = Donor_Activation_DF_2$GS1_Score ~
as.factor(Donor_Activation_DF_2$IV.Tobramycin..Y.N.))

Residuals:
    Min      1Q  Median      3Q     Max
-3.0499 -0.2438  0.3091  0.4349  1.7285

Coefficients:
                                                      Estimate Std. Error t value Pr(>|t|)
(Intercept)                                            12.7668     0.3532  36.144 1.98e-14 ***
as.factor(Donor_Activation_DF_2$IV.Tobramycin..Y.N.)Y  -1.8862     0.6840  -2.758   0.0163 *
---
Signif. codes:  0 '***' 0.001 '**' 0.01 '*' 0.05 '.' 0.1 ' ' 1

Residual standard error: 1.171 on 13 degrees of freedom
Multiple R-squared:  0.3691,    Adjusted R-squared:  0.3205
F-statistic: 7.605 on 1 and 13 DF,  p-value: 0.0163
```

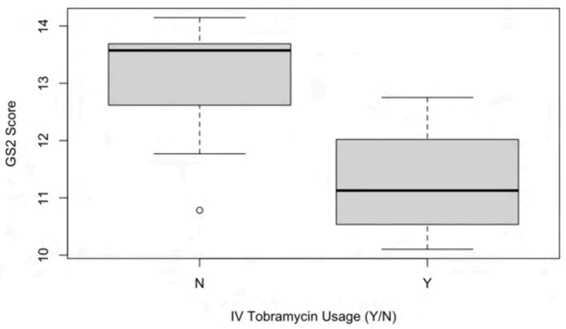


```
Call:
lm(formula = Donor_Activation_DF_2$GS2_Score ~
as.factor(Donor_Activation_DF_2$IV.Tobramycin..Y.N.))

Residuals:
     Min       1Q   Median       3Q      Max
-2.27731 -0.57422  0.08062  0.62991  1.47443

Coefficients:
                                                      Estimate Std. Error t value Pr(>|t|)
(Intercept)                                            13.0600     0.3202  40.785 4.17e-15 ***
as.factor(Donor_Activation_DF_2$IV.Tobramycin..Y.N.)Y  -1.7826     0.6201  -2.875    0.013 *
---
Signif. codes:  0 '***' 0.001 '**' 0.01 '*' 0.05 '.' 0.1 ' ' 1

Residual standard error: 1.062 on 13 degrees of freedom
Multiple R-squared:  0.3886,    Adjusted R-squared:  0.3416
F-statistic: 8.264 on 1 and 13 DF,  p-value: 0.01302
```

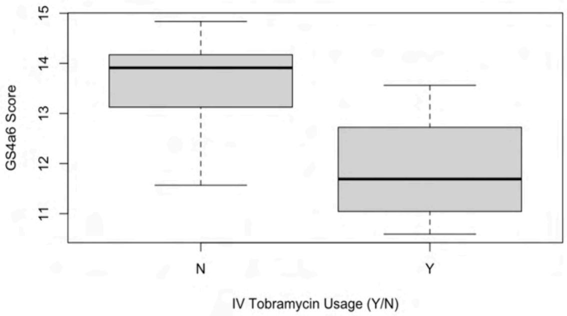


```
Call:
lm(formula = Donor_Activation_DF_2$GS4a6_Score ~
as.factor(Donor_Activation_DF_2$IV.Tobramycin..Y.N.))

Residuals:
     Min       1Q   Median       3Q      Max
-1.97397 -0.51331  0.08813  0.63075  1.67757

Coefficients:
                                                      Estimate Std. Error t value Pr(>|t|)
(Intercept)                                            13.5412     0.3256  41.586 3.25e-15 ***
as.factor(Donor_Activation_DF_2$IV.Tobramycin..Y.N.)Y  -1.6576     0.6306  -2.629   0.0208 *
---
Signif. codes:  0 '***' 0.001 '**' 0.01 '*' 0.05 '.' 0.1 ' ' 1

Residual standard error: 1.08 on 13 degrees of freedom
Multiple R-squared:  0.3471,    Adjusted R-squared:  0.2969
F-statistic:  6.91 on 1 and 13 DF,  p-value: 0.02084
```

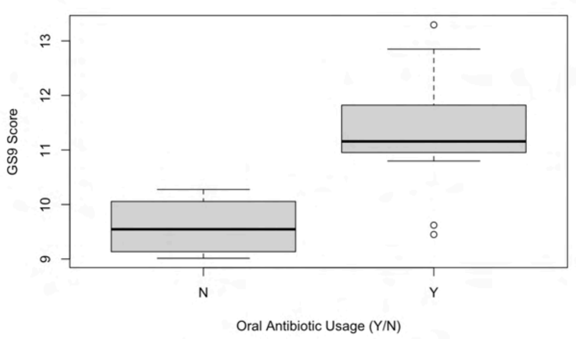


```
Call:
lm(formula = Donor_Activation_DF_2$GS9_Score ~ as.factor(Donor_Activation_DF_2$Oral.Abx..Y.N.))

Residuals:
    Min      1Q  Median      3Q     Max
-1.8453 -0.4183 -0.1372  0.4607  1.9994

Coefficients:
                                                 Estimate Std. Error t value Pr(>|t|)
(Intercept)                                        9.5952     0.5316  18.050 1.38e-10 ***
as.factor(Donor_Activation_DF_2$Oral.Abx..Y.N.)Y   1.6992     0.6208   2.737   0.0169 *
---
Signif. codes:  0 '***' 0.001 '**' 0.01 '*' 0.05 '.' 0.1 ' ' 1

Residual standard error: 1.063 on 13 degrees of freedom
Multiple R-squared:  0.3656,	Adjusted R-squared:  0.3168
F-statistic: 7.492 on 1 and 13 DF,  p-value: 0.01695
```

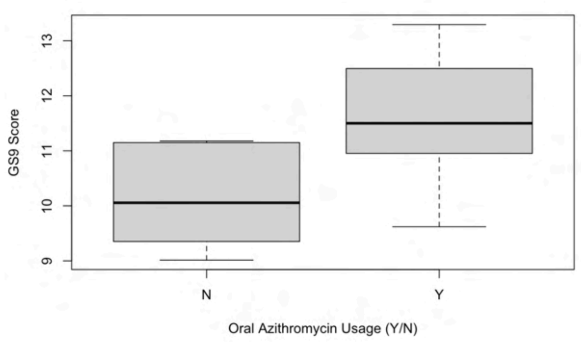


```
Call:
lm(formula = Donor_Activation_DF_2$GS9_Score ~
as.factor(Donor_Activation_DF_2$Oral.Azithromycin..Y.N.))

Residuals:
    Min      1Q  Median      3Q     Max
-1.9954 -0.7664 -0.1153  0.9858  1.6778

Coefficients:
                                                         Estimate Std. Error t value Pr(>|t|)
(Intercept)                                               10.1634     0.3834  26.509 1.06e-12
***
as.factor(Donor_Activation_DF_2$Oral.Azithromycin..Y.N.)Y   1.4526     0.5612   2.588   0.0225 *
---
Signif. codes:  0 '***' 0.001 '**' 0.01 '*' 0.05 '.' 0.1 ' ' 1

Residual standard error: 1.084 on 13 degrees of freedom
Multiple R-squared:  0.3401,	Adjusted R-squared:  0.2893
F-statistic: 6.699 on 1 and 13 DF,  p-value: 0.0225
```

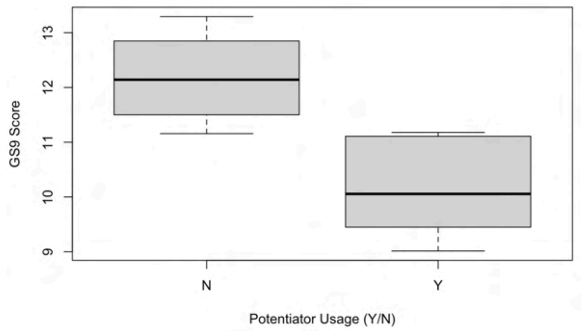


```
Call:
lm(formula = Donor_Activation_DF_2$GS9_Score ~
as.factor(Donor_Activation_DF_2$Potentiator.Therapy..Y.N.))

Residuals:
     Min       1Q   Median       3Q      Max
-1.15397 -0.70322 -0.04668  0.80066  1.10522

Coefficients:
                                                           Estimate Std. Error t value Pr(>|t|)
(Intercept)                                                 12.1886     0.3833  31.799 1.03e-13
***
as.factor(Donor_Activation_DF_2$Potentiator.Therapy..Y.N.)Y  -2.0210     0.4694  -4.305 0.000855
***
---
Signif. codes:  0 '***' 0.001 '**' 0.01 '*' 0.05 '.' 0.1 ' ' 1

Residual standard error: 0.8571 on 13 degrees of freedom
Multiple R-squared:  0.5877,	Adjusted R-squared:  0.556
F-statistic: 18.53 on 1 and 13 DF,  p-value: 0.0008552
```

Figure S6. Linear regression analysis demonstrates the association between metal concentration and the average expression of metal acquisition gene sets.

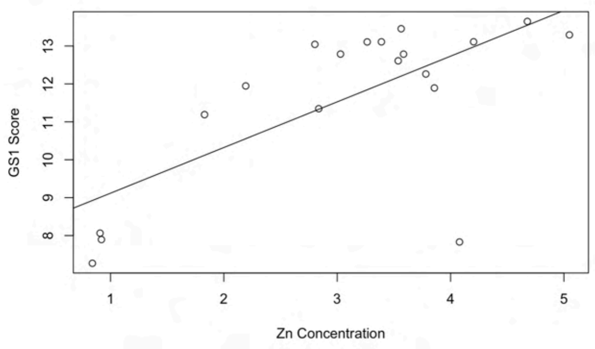


```
Call:
lm(formula = Media_Comparison_Metals$GS1_Score ~ Media_Comparison_Metals$Zn)

Residuals:
    Min      1Q  Median      3Q     Max 
-4.9961 -0.6843  0.1339  1.1684  1.7531 

Coefficients:
                           Estimate Std. Error t value Pr(>|t|)    
(Intercept)                  7.9133     1.0050   7.874 4.53e-07 ***
Media_Comparison_Metals$Zn   1.2046     0.3047   3.953  0.00103 ** 
---
Signif. codes:  0 ‘***’ 0.001 ‘**’ 0.01 ‘*’ 0.05 ‘.’ 0.1 ‘ ’ 1

Residual standard error: 1.598 on 17 degrees of freedom
Multiple R-squared:  0.479,	Adjusted R-squared:  0.4483 
F-statistic: 15.63 on 1 and 17 DF,  p-value: 0.001026
```

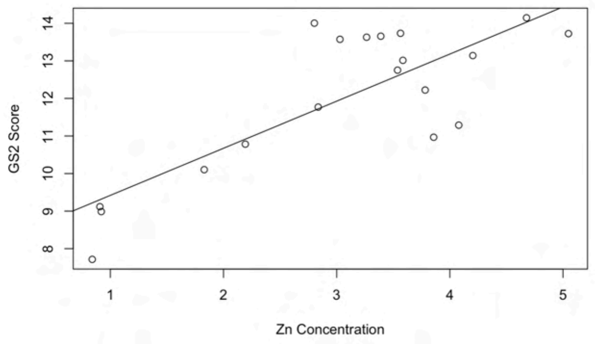


```
Call:
lm(formula = Media_Comparison_Metals$GS2_Score ~ Media_Comparison_Metals$Zn)

Residuals:
    Min      1Q  Median      3Q     Max 
-2.0368 -0.5217 -0.1331  0.7246  2.3230 

Coefficients:
                           Estimate Std. Error t value Pr(>|t|)    
(Intercept)                  8.1653     0.7563   10.80 4.98e-09 ***
Media_Comparison_Metals$Zn   1.2543     0.2293    5.47 4.15e-05 ***
---
Signif. codes:  0 ‘***’ 0.001 ‘**’ 0.01 ‘*’ 0.05 ‘.’ 0.1 ‘ ’ 1

Residual standard error: 1.203 on 17 degrees of freedom
Multiple R-squared:  0.6377,	Adjusted R-squared:  0.6164 
F-statistic: 29.92 on 1 and 17 DF,  p-value: 4.151e-05
```

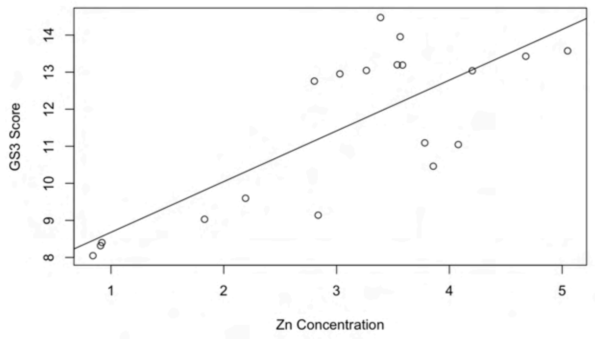


```
Call:
lm(formula = Media_Comparison_Metals$GS3_Score ~ Media_Comparison_Metals$Zn)

Residuals:
    Min      1Q  Median      3Q     Max 
-2.1277 -0.7489 -0.2346  1.1564  2.5241 

Coefficients:
                           Estimate Std. Error t value Pr(>|t|)    
(Intercept)                  7.3100     0.8886   8.226 2.49e-07 ***
Media_Comparison_Metals$Zn   1.3687     0.2694   5.080 9.27e-05 ***
---
Signif. codes:  0 ‘***’ 0.001 ‘**’ 0.01 ‘*’ 0.05 ‘.’ 0.1 ‘ ’ 1

Residual standard error: 1.413 on 17 degrees of freedom
Multiple R-squared:  0.6029,	Adjusted R-squared:  0.5795 
F-statistic: 25.81 on 1 and 17 DF,  p-value: 9.272e-05
```

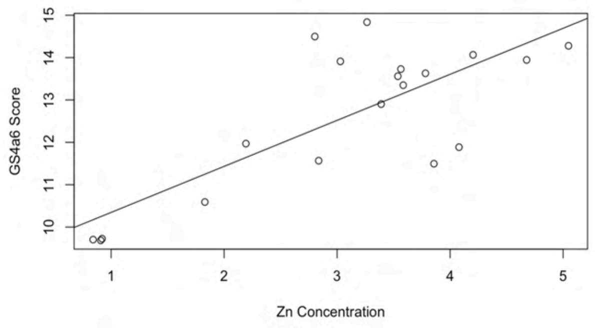


```
Call:
lm(formula = Media_Comparison_Metals$GS4a6_Score ~ Media_Comparison_Metals$Zn)

Residuals:
     Min       1Q   Median       3Q      Max 
-1.95488 -0.54731 -0.04055  0.39255  2.19248 

Coefficients:
                           Estimate Std. Error t value Pr(>|t|)    
(Intercept)                  9.2606     0.6977  13.273 2.12e-10 ***
Media_Comparison_Metals$Zn   1.0863     0.2115   5.135 8.26e-05 ***
---
Signif. codes:  0 ‘***’ 0.001 ‘**’ 0.01 ‘*’ 0.05 ‘.’ 0.1 ‘ ’ 1

Residual standard error: 1.109 on 17 degrees of freedom
Multiple R-squared:  0.608,	Adjusted R-squared:  0.585 
F-statistic: 26.37 on 1 and 17 DF,  p-value: 8.264e-05
```

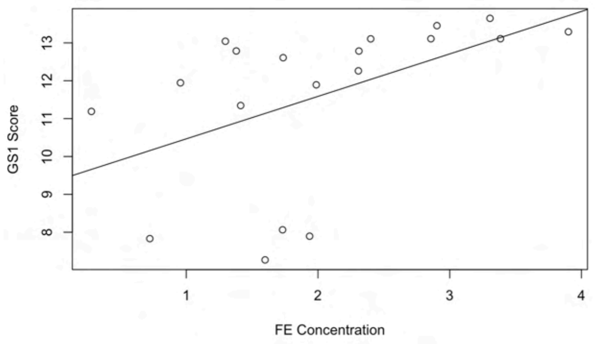


```
Call:
lm(formula = Media_Comparison_Metals$GS1_Score ~ Media_Comparison_Metals$Fe)

Residuals:
    Min      1Q  Median      3Q     Max 
-3.8692 -0.2305  0.5589  1.1934  2.2431 

Coefficients:
                           Estimate Std. Error t value Pr(>|t|)    
(Intercept)                  9.3445     1.0570   8.840 9.13e-08 ***
Media_Comparison_Metals$Fe   1.1220     0.4753   2.361   0.0304 *  
---
Signif. codes:  0 ‘***’ 0.001 ‘**’ 0.01 ‘*’ 0.05 ‘.’ 0.1 ‘ ’ 1

Residual standard error: 1.921 on 17 degrees of freedom
Multiple R-squared:  0.2469,	Adjusted R-squared:  0.2026 
F-statistic: 5.573 on 1 and 17 DF,  p-value: 0.03045
```

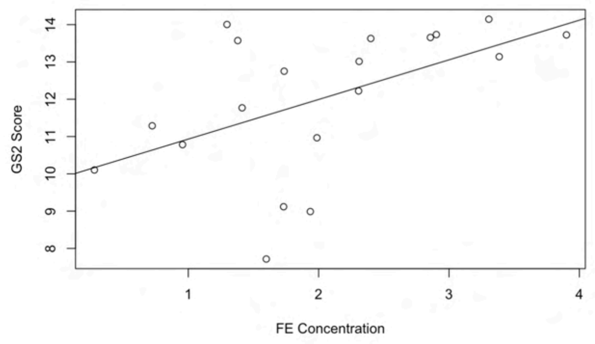


```
Call:
lm(formula = Media_Comparison_Metals$GS2_Score ~ Media_Comparison_Metals$Fe)

Residuals:
    Min      1Q  Median      3Q     Max 
-3.8520 -0.3082  0.3991  0.7713  2.7568 

Coefficients:
                           Estimate Std. Error t value Pr(>|t|)    
(Intercept)                  9.8686     0.9378   10.52 7.29e-09 ***
Media_Comparison_Metals$Fe   1.0629     0.4217    2.52    0.022 *  
---
Signif. codes:  0 ‘***’ 0.001 ‘**’ 0.01 ‘*’ 0.05 ‘.’ 0.1 ‘ ’ 1

Residual standard error: 1.705 on 17 degrees of freedom
Multiple R-squared:  0.272,	Adjusted R-squared:  0.2292 
F-statistic: 6.353 on 1 and 17 DF,  p-value: 0.022
```

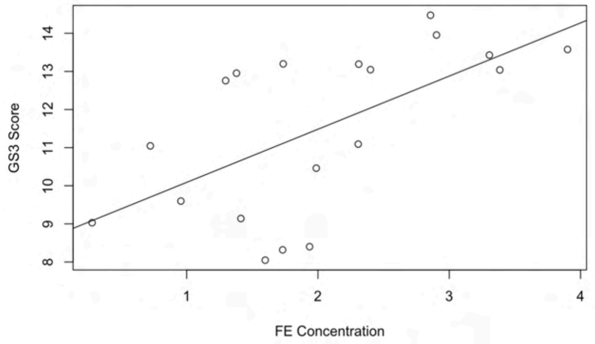


```
Call:
lm(formula = Media_Comparison_Metals$GS3_Score ~ Media_Comparison_Metals$Fe)

Residuals:
     Min       1Q   Median       3Q      Max 
-2.99267 -0.91103 -0.05395  1.30878  2.33509 

Coefficients:
                           Estimate Std. Error t value Pr(>|t|)    
(Intercept)                  8.6937     0.9776   8.893  8.4e-08 ***
Media_Comparison_Metals$Fe   1.3948     0.4396   3.173  0.00556 ** 
---
Signif. codes:  0 ‘***’ 0.001 ‘**’ 0.01 ‘*’ 0.05 ‘.’ 0.1 ‘ ’ 1

Residual standard error: 1.777 on 17 degrees of freedom
Multiple R-squared:  0.3719,	Adjusted R-squared:  0.335 
F-statistic: 10.07 on 1 and 17 DF,  p-value: 0.005562
```

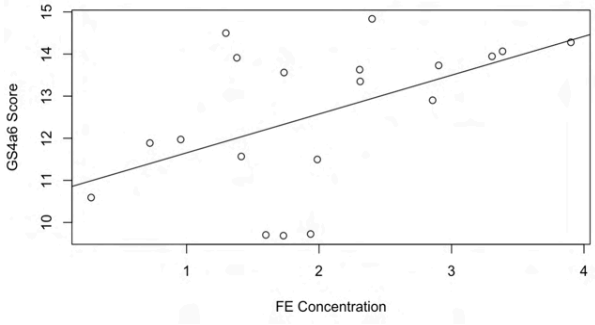


```
Call:
lm(formula = Media_Comparison_Metals$GS4a6_Score ~ Media_Comparison_Metals$Fe)

Residuals:
    Min      1Q  Median      3Q     Max 
-2.7926 -0.4666  0.2109  0.6284  2.5696 

Coefficients:
                           Estimate Std. Error t value Pr(>|t|)    
(Intercept)                 10.7318     0.8383  12.801 3.72e-10 ***
Media_Comparison_Metals$Fe   0.9225     0.3770   2.447   0.0256 *  
---
Signif. codes:  0 ‘***’ 0.001 ‘**’ 0.01 ‘*’ 0.05 ‘.’ 0.1 ‘ ’ 1

Residual standard error: 1.524 on 17 degrees of freedom
Multiple R-squared:  0.2605,	Adjusted R-squared:  0.217 
F-statistic: 5.989 on 1 and 17 DF,  p-value: 0.02556
```

Figure S7. Composition of RNA sequencing reads in the spike-in and ASM samples. Reads were mapped to various species including P. aeruginosa, other common CF pathogens, and the human genome.

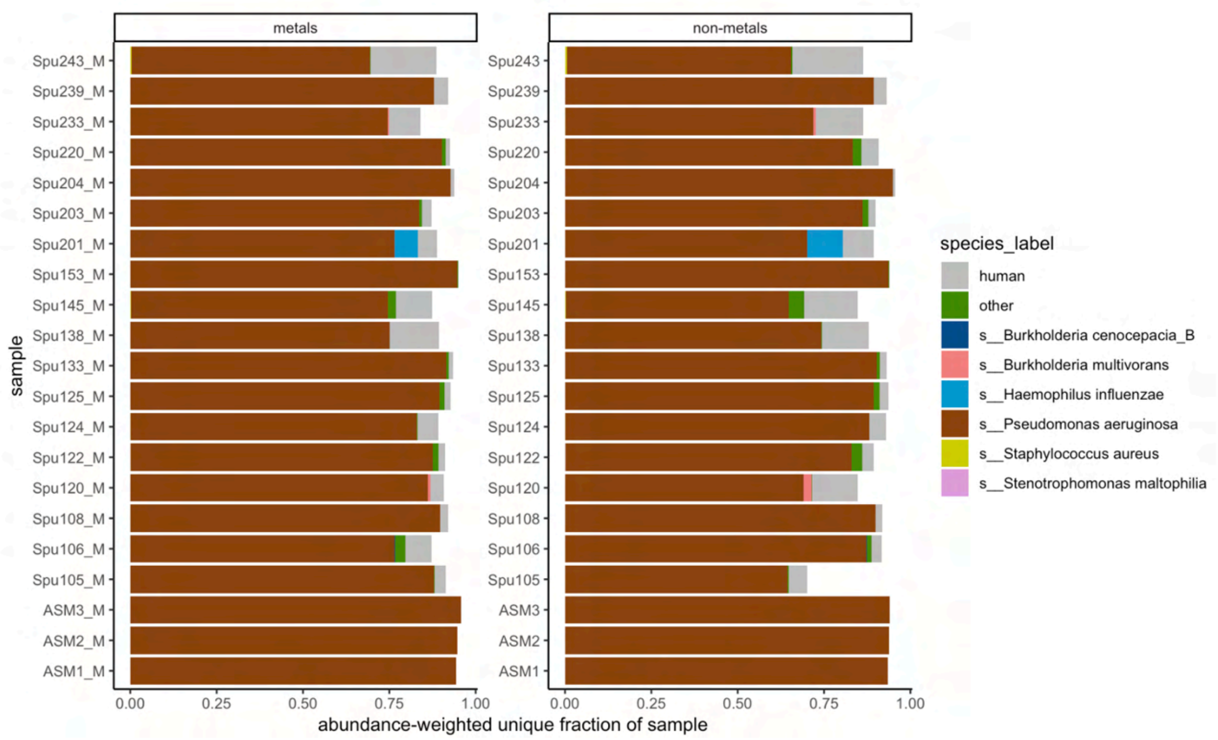

Figure S8. ADAGE-constructed gene sets were screened against an independent compendium of 890 PAO1 gene expression samples to check whether the constituent genes in each gene set remained correlated. All constituent genes in each gene set remain positively correlated, though some correlations are more moderate than others. The level of correlation is demonstrated in the correlation plots presented below.

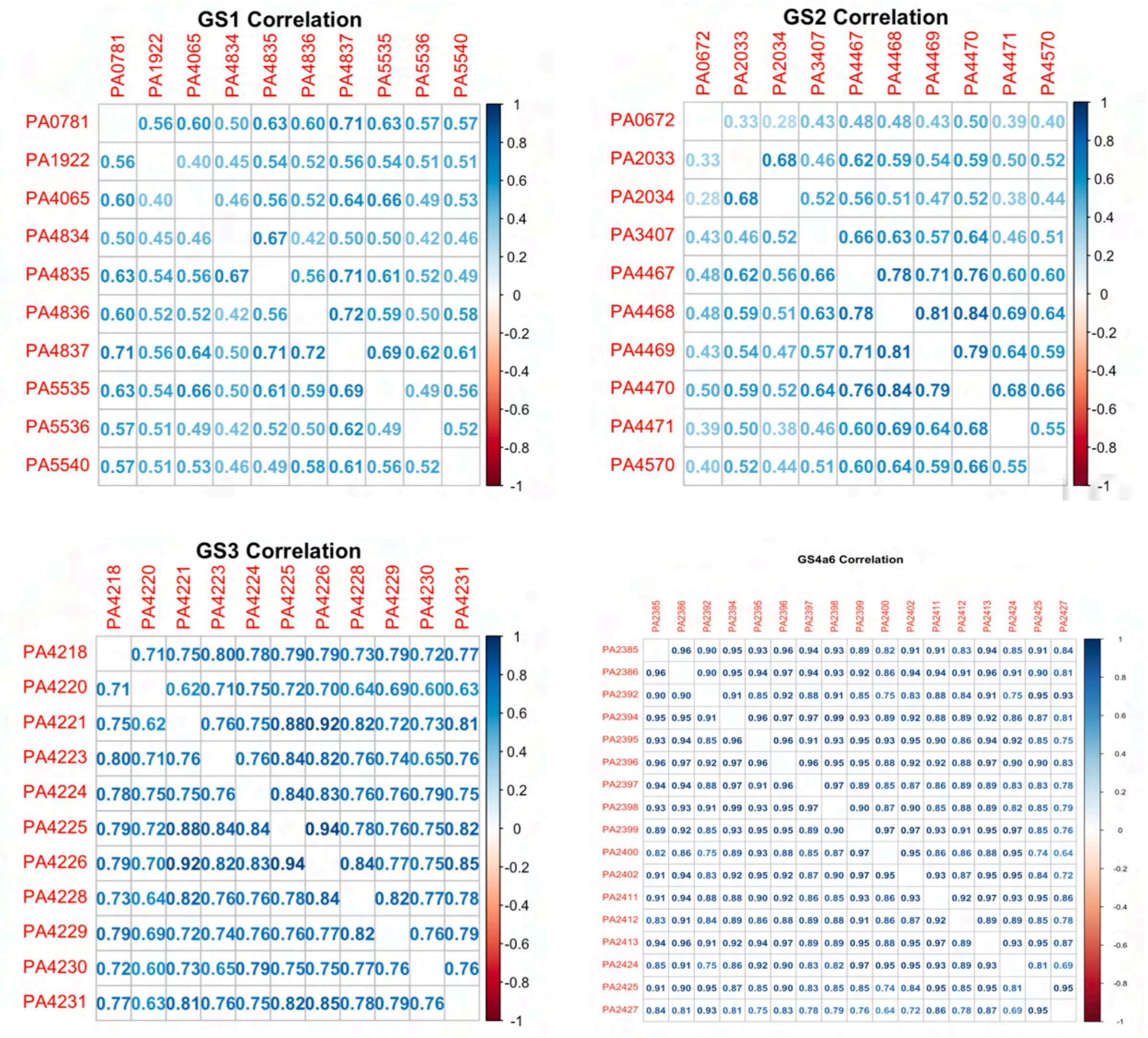

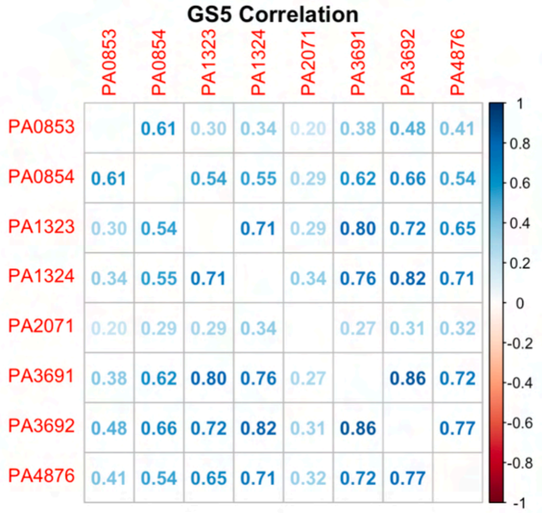
GS5 Correlation
PA0853
PA0854
PA1323
PA1324
PA2071
PA3691
PA3692
PA4876
1
0.8
0.6
0.4
0.2
0
-0.2
-0.4
-0.6
-0.8
-1

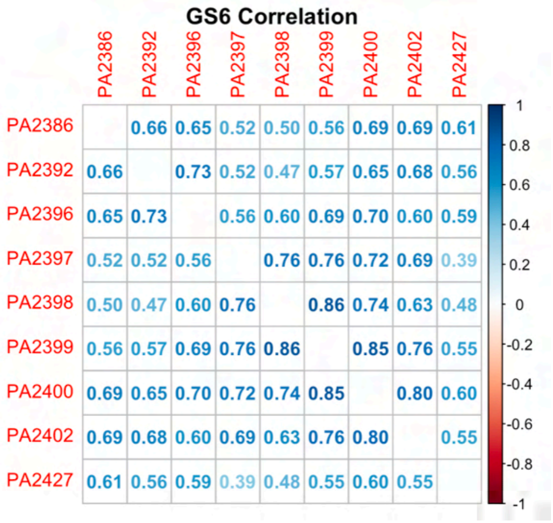
GS6 Correlation
PA2386
PA2392
PA2396
PA2397
PA2398
PA2399
PA2400
PA2402
PA2427

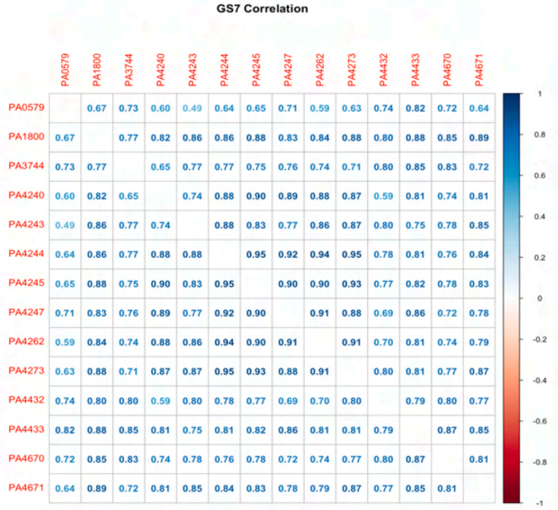
GS7 Correlation

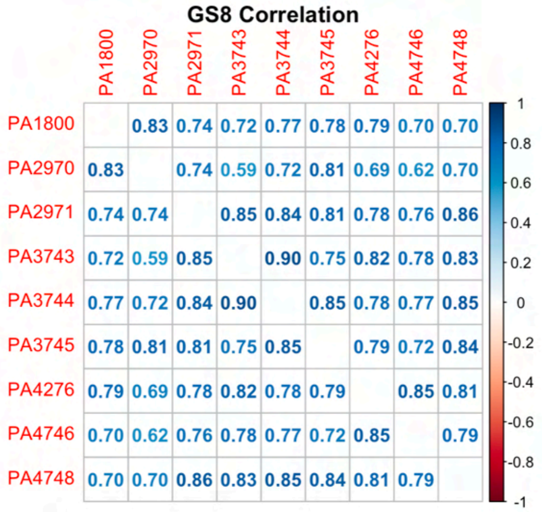
GS8 Correlation
PA1800
PA2970
PA2971
PA3743
PA3744
PA3745
PA4276
PA4746
PA4748

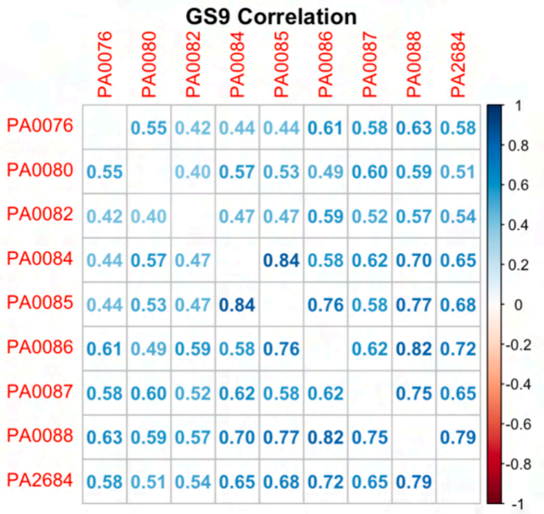
GS9 Correlation
PA0076
PA0080
PA0082
PA0084
PA0085
PA0086
PA0087
PA0088
PA2684

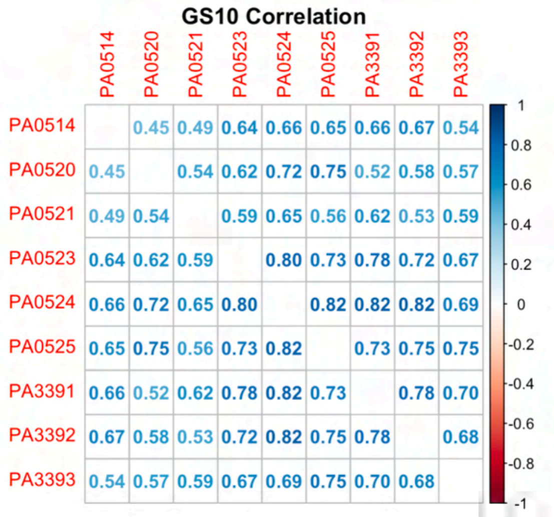
GS10 Correlation
PA0514
PA0520
PA0521
PA0523
PA0524
PA0525
PA3391
PA3392
PA3393

GS11 Correlation

	PA0767	PA2627	PA4449	PA4669
PA0767		0.41	0.68	0.71
PA2627	0.41		0.57	0.34
PA4449	0.68	0.57		0.53
PA4669	0.71	0.34	0.53	

GS12 Correlation

	PA3821	PA3822	PA3827	PA4480	PA4665	PA5568
PA3821		0.80	0.61	0.71	0.80	0.91
PA3822	0.80		0.73	0.69	0.63	0.79
PA3827	0.61	0.73		0.65	0.55	0.62
PA4480	0.71	0.69	0.65		0.78	0.75
PA4665	0.80	0.63	0.55	0.78		0.79
PA5568	0.91	0.79	0.62	0.75	0.79	

GS13 Correlation

	PA0506	PA2953	PA3006	PA3014
PA0506		0.44	0.64	0.72
PA2953	0.44		0.42	0.66
PA3006	0.64	0.42		0.46
PA3014	0.72	0.66	0.46	

GS14 Correlation

	PA0376	PA0456	PA1592	PA2738	PA2853	PA2966	PA4922	PA5253
PA0376		0.61	0.51	0.52	0.60	0.60	0.25	0.38
PA0456	0.61		0.45	0.47	0.75	0.72	0.36	0.50
PA1592	0.51	0.45		0.32	0.44	0.33	0.49	0.57
PA2738	0.52	0.47	0.32		0.61	0.71	0.37	0.20
PA2853	0.60	0.75	0.44	0.61		0.77	0.41	0.46
PA2966	0.60	0.72	0.33	0.71	0.77		0.40	0.23
PA4922	0.25	0.36	0.49	0.37	0.41	0.40		0.59
PA5253	0.38	0.50	0.57	0.20	0.46	0.23	0.59	

GS15 Correlation

	PA1777	PA2742	PA2743	PA4237	PA4265	PA4407	PA4922
PA1777		0.74	0.55	0.77	0.82	0.83	0.67
PA2742	0.74		0.85	0.73	0.80	0.61	0.43
PA2743	0.55	0.85		0.53	0.65	0.50	0.32
PA4237	0.77	0.73	0.53		0.86	0.61	0.48
PA4265	0.82	0.80	0.65	0.86		0.75	0.59
PA4407	0.83	0.61	0.50	0.61	0.75		0.67
PA4922	0.67	0.43	0.32	0.48	0.59	0.67	

GS16 Correlation

	PA0779	PA1596	PA3126	PA4061	PA4385	PA4386	PA4542	PA4760	PA4761	PA4762	PA5053	PA5054
PA0779		0.86	0.66	0.74	0.74	0.71	0.86	0.80	0.87	0.80	0.84	0.87
PA1596	0.86		0.65	0.78	0.86	0.82	0.80	0.76	0.83	0.77	0.82	0.82
PA3126	0.66	0.65		0.55	0.50	0.39	0.69	0.55	0.65	0.54	0.61	0.61
PA4061	0.74	0.78	0.55		0.70	0.66	0.69	0.64	0.69	0.65	0.79	0.71
PA4385	0.74	0.86	0.50	0.70		0.91	0.70	0.72	0.83	0.71	0.68	0.74
PA4386	0.71	0.82	0.39	0.66	0.91		0.67	0.70	0.79	0.79	0.70	0.73
PA4542	0.86	0.80	0.69	0.69	0.70	0.67		0.82	0.87	0.77	0.78	0.80
PA4760	0.80	0.76	0.55	0.64	0.72	0.70	0.82		0.89	0.78	0.75	0.83
PA4761	0.87	0.83	0.65	0.69	0.83	0.79	0.87	0.89		0.85	0.80	0.91
PA4762	0.80	0.77	0.54	0.65	0.71	0.79	0.77	0.78	0.85		0.85	0.88
PA5053	0.84	0.82	0.61	0.79	0.68	0.70	0.78	0.75	0.80	0.85		0.90
PA5054	0.87	0.82	0.61	0.71	0.74	0.73	0.80	0.83	0.91	0.88	0.90	

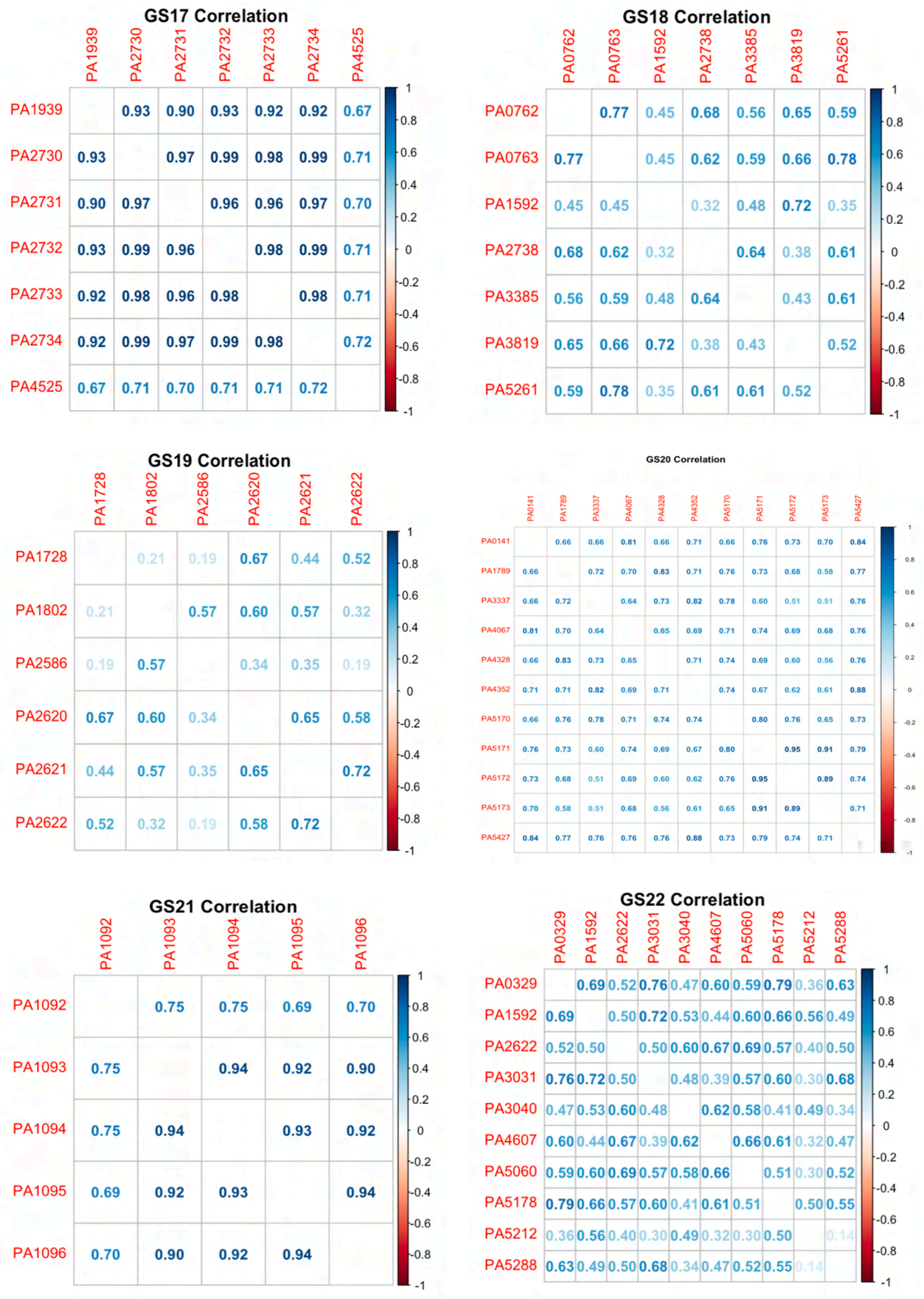
GS17 Correlation
PA1939 PA2730 PA2731 PA2732 PA2733 PA2734 PA4525
PA1939 0.93 0.90 0.93 0.92 0.92 0.67
PA2730 0.93 0.97 0.99 0.98 0.99 0.71
PA2731 0.90 0.97 0.96 0.96 0.97 0.70
PA2732 0.93 0.99 0.96 0.98 0.99 0.71
PA2733 0.92 0.98 0.96 0.98 0.98 0.71
PA2734 0.92 0.99 0.97 0.99 0.98 0.72
PA4525 0.67 0.71 0.70 0.71 0.71 0.72
1 0.8 0.6 0.4 0.2 0 -0.2 -0.4 -0.6 -0.8 -1
GS18 Correlation
PA0762 PA0763 PA1592 PA2738 PA3385 PA3819 PA5261
PA0762 0.77 0.45 0.68 0.56 0.65 0.59
PA0763 0.77 0.45 0.62 0.59 0.66 0.78
PA1592 0.45 0.45 0.32 0.48 0.72 0.35
PA2738 0.68 0.62 0.32 0.64 0.38 0.61
PA3385 0.56 0.59 0.48 0.64 0.43 0.61
PA3819 0.65 0.66 0.72 0.38 0.43 0.52
PA5261 0.59 0.78 0.35 0.61 0.61 0.52
1 0.8 0.6 0.4 0.2 0 -0.2 -0.4 -0.6 -0.8 -1
GS19 Correlation
PA1728 PA1802 PA2586 PA2620 PA2621 PA2622
PA1728 0.21 0.19 0.67 0.44 0.52
PA1802 0.21 0.57 0.60 0.57 0.32
PA2586 0.19 0.57 0.34 0.35 0.19
PA2620 0.67 0.60 0.34 0.65 0.58
PA2621 0.44 0.57 0.35 0.65 0.72
PA2622 0.52 0.32 0.19 0.58 0.72
1 0.8 0.6 0.4 0.2 0 -0.2 -0.4 -0.6 -0.8 -1
GS20 Correlation
PA0141 PA1789 PA3337 PA4067 PA4328 PA4352 PA5170 PA5171 PA5172 PA5173 PA5427
PA0141 0.66 0.66 0.81 0.66 0.71 0.66 0.76 0.73 0.70 0.84
PA1789 0.66 0.72 0.70 0.83 0.71 0.76 0.73 0.68 0.58 0.77
PA3337 0.66 0.72 0.64 0.73 0.82 0.78 0.60 0.51 0.51 0.76
PA4067 0.81 0.70 0.64 0.65 0.69 0.71 0.74 0.69 0.68 0.76
PA4328 0.66 0.83 0.73 0.65 0.71 0.74 0.69 0.60 0.56 0.76
PA4352 0.71 0.71 0.82 0.69 0.71 0.74 0.67 0.62 0.61 0.88
PA5170 0.66 0.76 0.78 0.71 0.74 0.74 0.80 0.76 0.65 0.73
PA5171 0.76 0.73 0.60 0.74 0.69 0.67 0.80 0.95 0.91 0.79
PA5172 0.73 0.68 0.51 0.69 0.60 0.62 0.76 0.95 0.89 0.74
PA5173 0.70 0.58 0.51 0.68 0.56 0.61 0.65 0.91 0.89 0.71
PA5427 0.84 0.77 0.76 0.76 0.76 0.88 0.73 0.79 0.74 0.71
1 0.8 0.6 0.4 0.2 0 -0.2 -0.4 -0.6 -0.8 -1
GS21 Correlation
PA1092 PA1093 PA1094 PA1095 PA1096
PA1092 0.75 0.75 0.69 0.70
PA1093 0.75 0.94 0.92 0.90
PA1094 0.75 0.94 0.93 0.92
PA1095 0.69 0.92 0.93 0.94
PA1096 0.70 0.90 0.92 0.94
1 0.8 0.6 0.4 0.2 0 -0.2 -0.4 -0.6 -0.8 -1
GS22 Correlation
PA0329 PA1592 PA2622 PA3031 PA3040 PA4607 PA5060 PA5178 PA5212 PA5288
PA0329 0.69 0.52 0.76 0.47 0.60 0.59 0.79 0.36 0.63
PA1592 0.69 0.50 0.72 0.53 0.44 0.60 0.66 0.56 0.49
PA2622 0.52 0.50 0.50 0.60 0.67 0.69 0.57 0.40 0.50
PA3031 0.76 0.72 0.50 0.48 0.39 0.57 0.60 0.30 0.68
PA3040 0.47 0.53 0.60 0.48 0.62 0.58 0.41 0.49 0.34
PA4607 0.60 0.44 0.67 0.39 0.62 0.66 0.61 0.32 0.47
PA5060 0.59 0.60 0.69 0.57 0.58 0.66 0.51 0.30 0.52
PA5178 0.79 0.66 0.57 0.60 0.41 0.61 0.51 0.50 0.55
PA5212 0.36 0.56 0.40 0.30 0.49 0.32 0.30 0.50 0.14
PA5288 0.63 0.49 0.50 0.68 0.34 0.47 0.52 0.55 0.14
1 0.8 0.6 0.4 0.2 0 -0.2 -0.4 -0.6 -0.8 -1

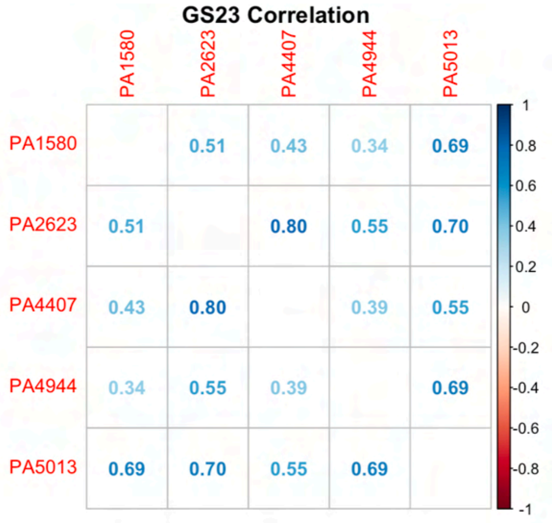
GS23 Correlation
PA1580
PA2623
PA4407
PA4944
PA5013
PA1580 0.51 0.43 0.34 0.69
PA2623 0.51 0.80 0.55 0.70
PA4407 0.43 0.80 0.39 0.55
PA4944 0.34 0.55 0.39 0.69
PA5013 0.69 0.70 0.55 0.69
1
0.8
0.6
0.4
0.2
0
-0.2
-0.4
-0.6
-0.8
-1

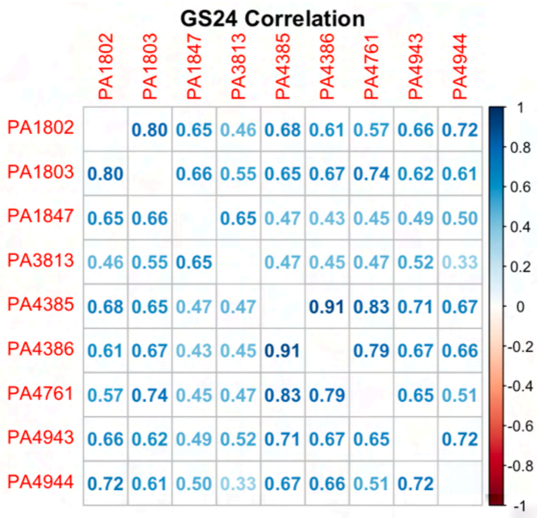
GS24 Correlation
PA1802
PA1803
PA1847
PA3813
PA4385
PA4386
PA4761
PA4943
PA4944
PA1802 0.80 0.65 0.46 0.68 0.61 0.57 0.66 0.72
PA1803 0.80 0.66 0.55 0.65 0.67 0.74 0.62 0.61
PA1847 0.65 0.66 0.65 0.47 0.43 0.45 0.49 0.50
PA3813 0.46 0.55 0.65 0.47 0.45 0.47 0.52 0.33
PA4385 0.68 0.65 0.47 0.47 0.91 0.83 0.71 0.67
PA4386 0.61 0.67 0.43 0.45 0.91 0.79 0.67 0.66
PA4761 0.57 0.74 0.45 0.47 0.83 0.79 0.65 0.51
PA4943 0.66 0.62 0.49 0.52 0.71 0.67 0.65 0.72
PA4944 0.72 0.61 0.50 0.33 0.67 0.66 0.51 0.72
1
0.8
0.6
0.4
0.2
0
-0.2
-0.4
-0.6
-0.8
-1
